# Metabolite and thymocyte development defects in ADA-SCID mice receiving enzyme replacement therapy

**DOI:** 10.1038/s41598-021-02572-w

**Published:** 2021-12-01

**Authors:** Federico A. Moretti, Giuliana Giardino, Teresa C. H. Attenborough, Athina Soragia Gkazi, Ben K. Margetts, Giancarlo la Marca, Lynette Fairbanks, Tessa Crompton, H. Bobby Gaspar

**Affiliations:** 1 UCL Great Ormond Street Institute of Child Health, London, UK; 2 Department of Experimental and Clinical Biomedical Sciences, University of Florence and Newborn Screening, Clinical Chemistry and Pharmacology Lab, Meyer Children’s Hospital, Florence, Italy; 3 Purine Research Laboratory, St Thomas’ Hospital, London, UK

**Keywords:** Apoptosis, Mechanisms of disease, Mass spectrometry, Primary immunodeficiency disorders, Severe combined immunodeficiency, Lymphocytes, Thymus

## Abstract

Deficiency of adenosine deaminase (ADA, EC3.5.4.4), a housekeeping enzyme intrinsic to the purine salvage pathway, leads to severe combined immunodeficiency (SCID) both in humans and mice. Lack of ADA results in the intracellular accumulation of toxic metabolites which have effects on T cell development and function. While untreated ADA-SCID is a fatal disorder, there are different therapeutic options available to restore ADA activity and reconstitute a functioning immune system, including enzyme replacement therapy (ERT). Administration of ERT in the form of pegylated bovine ADA (PEG-ADA) has proved a life-saving though non-curative treatment for ADA-SCID patients. However, in many patients treated with PEG-ADA, there is suboptimal immune recovery with low T and B cell numbers. Here, we show reduced thymus cellularity in ADA-SCID mice despite weekly PEG-ADA treatment. This was associated with lack of effective adenosine (Ado) detoxification in the thymus. We also show that thymocyte development in ADA-deficient thymi is arrested at the DN3-to-DN4 stage transition with thymocytes undergoing dATP-induced apoptosis rather than defective TCRβ rearrangement or β-selection. Our studies demonstrate at a detailed level that exogenous once-a-week enzyme replacement does not fully correct intra-thymic metabolic or immunological abnormalities associated with ADA deficiency.

## Introduction

Adenosine deaminase-deficient severe combined immunodeficiency (ADA-SCID) is a primary immunodeficiency characterized by severe pan-lymphopenia (T^−^, B^−^ and NK^−^), severe and recurrent infections, failure to thrive and death in the first year of life, if left untreated^1,2^. Lack of ADA leads to increased intra- and extracellular accumulation of adenosine (Ado) and deoxyadenosine (dAdo), and increased intracellular conversion of dAdo to deoxyadenosine triphosphate (dATP), thus expanding both dATP and Ado pools^3^. High levels of dATP are thought to be the main cause of lymphotoxicity, leading to increased thymic apoptosis^4^, defective DNA replication and repair^5^, altered V(D)J recombination and antigen receptor diversity^6^, and altered T and B cell receptor (TCR and BCR) signaling^7,8^.

A murine model, generated using a two-stage genetic engineering strategy^9^, has been shown to recapitulate many features associated with ADA deficiency in humans, including combined immunodeficiency and death 3 weeks after birth, if left untreated^10^. The severe lymphopenia in these mice is also associated to a pronounced accumulation of Ado, dAdo/dATP in thymus and spleen^9^.

Treatment options for ADA-SCID which have successfully restored lymphocyte development and function in patients are limited to allogeneic hematopoietic stem cell (HSC) transplantation (HSC-T)^11^, Enzyme replacement therapy (ERT) by twice-a-week injections of pegylated ADA (PEG-ADA)^12^ and more recently gene therapy (GT) using genetically modified autologous HSC (HSC-GT)^13^.

PEG-ADA acts exogenously and deaminates only the extracellular Ado and dAdo pools, thereby generating a concentration gradient which draws these metabolites out of the intracellular compartments and induces a rapid effective metabolic detoxification, thus improving patient clinical well-being. However, both in short and long-term ERT, the immune recovery in most patients remains suboptimal with reduced lymphocyte counts, low T cell numbers despite initial improvement and poor thymic output as measured by T-cell receptor excision circle (TREC) levels and naïve T cell markers^14,15^. The reasons for poor thymic and T cell recovery following PEG-ADA remain unclear, especially since alloHSC-T^16^ and autologous HSC-GT^17,18^ both show effective reconstitution of thymopoiesis.

To address this issue, we investigated and monitored the immune recovery in ADA-deficient mice that have been treated with PEG-ADA for up to a year. As expected, these mice were rescued from death and were viable and fertile. However, despite normal peripheral lymphocyte counts and normalization of ADA metabolites in peripheral blood, thymus size and cellularity remained significantly reduced compared to controls. We also noticed ongoing abnormalities of Ado detoxification despite normalization of dAdo and dATP levels in ERT thymi.

This study clearly demonstrates that extracellular supply of ADA enzyme through ERT results in ongoing metabolic abnormalities and associated defect in thymopoiesis, that are reflective of the suboptimal immune recovery in patients with ADA deficiency receiving long-term ERT.

## Results

### Loss of ADA leads to progressive thymus atrophy and severe lymphopenia

**Figure 1:**
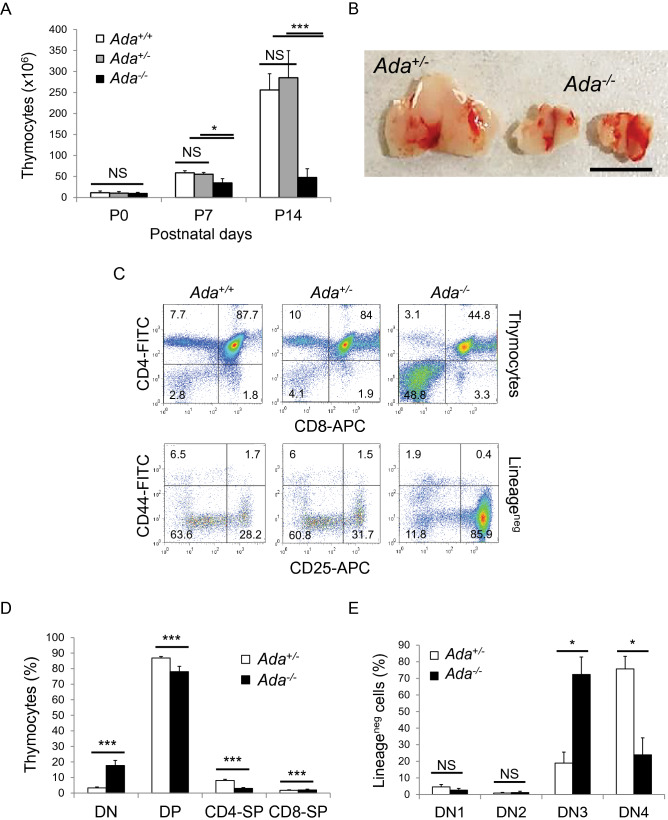
ADA-deficient mice show severe thymus atrophy and impaired T cell development. (**A**) Thymus cellularity at birth (P0) (n = 4, 5, 4), at P7 (n = 3, 3, 3) and at P14 (n = 9, 9, 12). (**B**) In vivo images of thymi taken immediately after dissection at P14. (**C**) FACS plots of thymocytes from P14 thymi showing (upper panel) cell distribution among DN (CD4^*−*^, CD8^*−*^), DP (CD4^+^, CD8^+^), CD4-SP (CD4^+^, CD8^*−*^) and CD8-SP (CD4^*−*^, CD8^+^) populations and (lower panel) cell distribution among DN1 (CD44^+^, CD25^*−*^), DN2 (CD44^+^, CD25^+^), DN3 (CD44^*−*^, CD25^+^) and DN4 (CD44^*−*^, CD25^*−*^) Lin^neg^ populations. (**D** and **E**) Bar graphs representing percentages of cell population shown in (C) (n = 6, 4). DN = double negative, DP = double positive, SP = single positive. Quadrant numbers represent relative cell percentages. Statistical analysis was performed using the two-tailed homoscedastic Student’s t-Test. Data represent means ± SD. *P* values < 0.05 were considered significant. *P < 0.05, **P < 0.01 and ***P < 0.001. NS = statistically not significant.

We first investigated and compared thymus size and cellularity in *Ada* wild-type (*Ada*^+*/*+^) mice and *Ada* heterozygous (*Ada*^+*/−*^) and homozygous (*Ada*^*−/−*^) mutant mice. Thymi dissected from *Ada*^+*/−*^ mice did not show significant differences in size and cellularity (Fig. 1A) compared to *Ada*^+*/*+^ thymi, despite a 50% reduction in ADA expression levels (Supplementary Fig. 1A, B). In contrast, ADA-deficient thymi dissected from *Ada*^*−/−*^ mice (Supplementary Fig. 1A) showed a progressive and significant postnatal reduction in thymocyte numbers compared to *Ada*^+*/*+^ and *Ada*^+*/−*^ control thymi (Fig. 1A,B). At 1 week after birth, postnatal day (P) 7, cell numbers in *Ada*^*−/−*^ thymi were reduced to ~ 60% of control levels and to ~ 18% at 2 weeks of age (P14) (Fig. 1A,B). It is worth noting that at birth (P0), thymocyte numbers were comparable among the three genotypes (*Ada*^+*/*+^, *Ada*^+*/−*^ and *Ada*^*−/−*^), suggesting relatively normal thymic development during fetal life.

We next used tandem mass spectrometry (MS) to quantify content of Ado and dAdo and reversed phase high-performance liquid chromatography (HPLC) to measure dATP content in extracts of thymus and blood from 2-week-old *Ada*^+*/*+^, *Ada*^+*/−*^ and *Ada*^*−/−*^ mice. As expected, levels of Ado, dAdo and dATP were significantly increased in *Ada*^*−/−*^ thymus and blood compared to *Ada*^+*/*+^ and *Ada*^+*/−*^ control tissues (Supplementary Fig. 1C-F). Severe thymus atrophy in the *Ada*^*−/−*^ mice was accompanied by severe T cell lymphopenia in blood (Supplementary Fig. 2A,B) and spleen (Supplementary Fig. 3A-C).

These data clearly confirm the profound immunologic disturbances in mice with abrogated ADA expression and mirror the defects seen in untreated ADA-deficient patients.

### Lack of ADA blocks thymocyte development at the DN3 stage

Since peripheral T cells were reduced in *Ada*^*−/−*^ mice, we examined the different stages of T cell development within the thymus. Thymi from *Ada*^*−/−*^ mice at P14 presented an abnormal distribution of thymocytes among the four thymic subpopulations by FACS staining using anti-CD4 and anti-CD8 antibodies (Ab) (Fig. 1C). We found an elevated percentage of the CD4^*−*^/CD8^*−*^ double negative (DN) cells and, consequently, a reduced frequency of both CD4^+^/CD8^+^ double positive (DP) and of CD4^+^ and CD8^+^ single positive (SP) cells (Fig. 1D). Due to the overall reduced cellularity of *Ada*^*−/−*^ thymi, the absolute number of the DP cells was significantly reduced and the SP cells were nearly absent compared to control cells (Supplementary Fig. 4A). DN cells, instead, were present in similar numbers to control thymi, consistent with the highly elevated percentage of the DN cell population in the *Ada*^*−/−*^ thymi (*Ada*^*−/−*^ = 23.34% ± 14.69) compared to control DN cells (*Ada*^+*/−*^ = 3.57% ± 0.66) (Fig. 1D, Supplementary Fig. 4A). These experiments clearly indicated that thymocytes fail to develop beyond the DN stage in the absence of ADA.

To define further the stage at which thymocyte development is blocked in *Ada*^*−/−*^ thymi, we identified the four discrete DN subsets of the thymus lineage negative (Lin^neg^) population by FACS analysis based on expression of CD44 and CD25 receptors: DN1 (Lin^neg^, CD44^+^, CD25^*−*^), DN2 (Lin^neg^, CD44^+^, CD25^+^), DN3 (Lin^neg^, CD44^*−*/lo^, CD25^+^) and DN4 (Lin^neg^, CD44^*−*/lo^, CD25^*−*^)^19,20^. Thymocyte development is arrested at the DN3 stage in *Ada*^*−/−*^ deficient animals (Fig. 1C). Indeed, the number and proportion of the DN3 subset was significantly greater in the *Ada*^*−/−*^ thymus compared with control thymus, whereas the DN4 subpopulation was significantly reduced (Fig. 1E, Supplementary Fig. 4B). The DN1 and DN2 populations were normal in the *Ada*^*−/−*^ thymus, suggesting that the DN3 cells are specifically affected by the absence of ADA expression.

H&E staining of paraffin-embedded sections of thymi from P14 mice showed altered tissue architecture in the *Ada*^*−/−*^ thymus with no well-defined cortico-medullary boundaries in comparison to control thymi (Supplementary Fig. 4C). This is very likely due to thymic atrophy and the absence of both CD4^+^ and CD8^+^ SP cells, which are normally located in the central medulla.

Taken together, these data suggest that the atrophy in the *Ada*^*−/−*^ thymus is due to the inability of thymocytes to develop beyond the DN3 (CD44^*−*/lo^, CD25^+^) subset of the CD4^*−*^CD8^*−*^ DN stage.

### Reduced production of BM-derived hematopoietic progenitor cells in Ada^−/−^ mice

**Figure 2:**
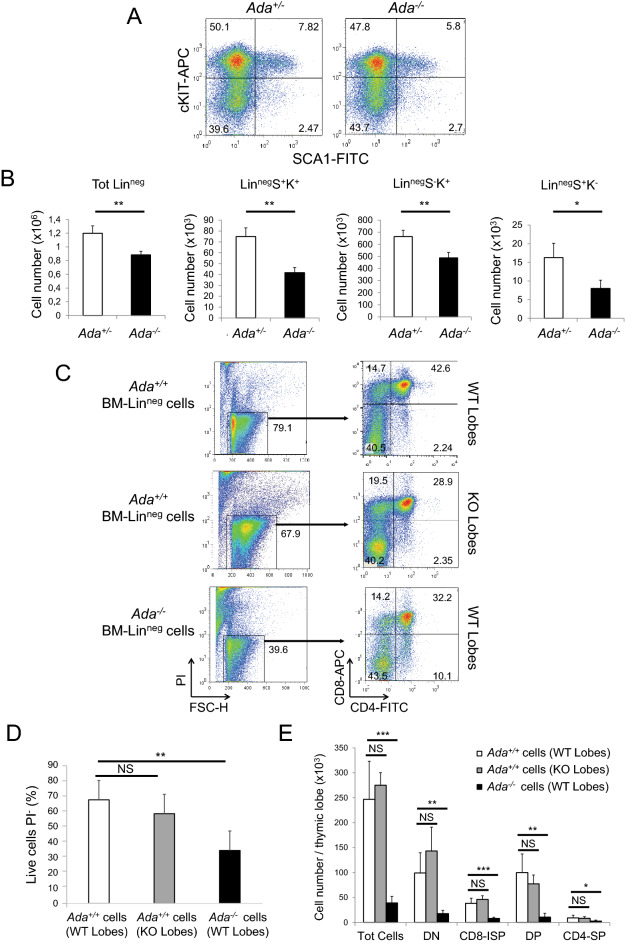
BM-derived HSPC populations from ADA-deficient mice show reduced cellularity and impaired development and function. (**A**) FACS plots of BM Lin^neg^ cell populations from mice at P14: primitive hematopoietic stem cells (SCA1^+^, cKIT^+^), myeloid progenitor cells (SCA1^*−*^, cKIT^+^) and early lymphoid-committed precursors (SCA1^+^, cKIT^*−*^). (**B**) Bar graphs representing absolute numbers of cell populations shown in (A) per mouse (n = 4, 3). (**C**) FTOC performed coculturing thymic lobes with BM Lin^neg^ cells for 3 weeks. FACS plots show live cells (PI negative populations) recovered from squeezed lobes (left panels) and distribution of developing thymocytes (right panels). Immature CD8-SP (CD8-ISP) (CD4^*−*^, CD8^+^), DP (CD4^+^, CD8^+^), CD4-SP (CD4^+^, CD8^*−*^). (**D**) Bar graph representing percentage of live cells shown in (C) (n = 6, 4, 5). (**E**) Total number of cells per thymic lobe per each experiment condition (n = 6, 4, 5). *P < 0.05, **P < 0.01 and ***P < 0.001. NS = statistically not significant.

We next assessed whether loss of ADA expression affected production of hematopoietic stem and progenitor cells (HSPCs) by the bone marrow (BM). We isolated BM cells from *Ada*^*−/−*^ and control femurs and after magnetic depletion of mature hematopoietic cells, the Lin^neg^ population was analyzed by FACS for surface expression of cKIT (CD117) and SCA1 (Fig. 2A), allowing us to quantify cells of each HSPC population: (1) early lymphoid-committed precursors (Lin^neg^, SCA1^+^, cKIT^*−*^)^21^, (2) myeloid progenitor population (Lin^neg^, SCA1^*−*^, cKIT^+^)^22^ and (3) primitive hematopoietic stem cells (Lin^neg^, SCA1^+^, cKIT^+^)^23^. The cell number of the Lin^neg^ cell population and of each progenitor population was significantly reduced in *Ada*^*−/−*^ BM compared to controls (Fig. 2B).

This observation clearly showed the deleterious effect of the lack of ADA expression in the development or maintenance of HSPC in *Ada*^*−/−*^ animals and may play a role in the seeding of progenitor populations to the thymus in untreated mice.

### Loss of ADA expression does not affect thymic stromal cell function

An impaired ability of *Ada*^*−/−*^ thymic stromal cells (TSCs) to support T cell development may be an additional problem contributing to thymic atrophy^11^. To test this, we established fetal thymus organ cultures (FTOC)^24^ coculturing both *Ada*^+*/*+^ (WT) and *Ada*^*−/−*^ (KO) 2-deoxyguanosine (dGuo)-treated alymphoid thymic lobes^25^ from 15-day-old embryos (E15) with BM-derived HSPCs. After 3 weeks, intrathymic T cell development was verified by FACS analysis using antibodies against CD4 and CD8 (Fig. 2C). *Ada*^+*/*+^ BM-derived Lin^neg^ cells were equally able to colonize both WT and KO lobes and generate T cells, suggesting that accumulation of toxic metabolites within *Ada*^*−/−*^ TSCs does not interfere with their development, maturation and ability to support T cell development (Fig. 2D,E). In contrast, *Ada*^*−/−*^ BM-derived Lin^neg^ cells, even when grown on *Ada*^+*/*+^ TSCs from WT lobes, showed reduced viability (Fig. 2D) and severely impaired T cell differentiation (Fig. 2E), thus suggesting that the thymic development defect in *Ada*^*−/−*^ mice is a T cell progenitor-intrinsic abnormality rather than a failure of appropriate support from the thymic stromal epithelium.

### The nucleotide pool imbalance does not induce alterations of N-region insertions during TCRβ chain V(D)J recombination

Considering that thymocyte development was arrested at DN3 stage, we investigated whether ADA deficiency could impair the pre-TCR checkpoint. We firstly evaluated the intracellular and surface expression of TCRβ in DN3 and DN4 thymocytes. No difference was observed between *Ada*^*−/−*^ and *Ada*^+*/−*^ thymocytes in both the stages, suggesting that ADA deficiency does not affect TCRβ expression (Supplementary Fig. 5A,B).

We next examined whether V(D)J recombination and N-region additions were affected by the absence of ADA and the consequent metabolic disturbance of dAdo/dATP levels (Supplementary Fig. 1C,D). The TCRβ chain is first expressed in DN3 thymocytes (Lin^neg^, CD44^*−*/lo^, CD25^+^) upon successful rearrangement of a TCRβ gene. Association of TCRβ chain with the surrogate α chain (pre-Tα) and the CD3 subunits results in expression of the pre-TCR complex at the DN3 stage. Signaling through the pre-TCR complex at the DN3 stage drives differentiation of these cells to the DN4 (Lin^neg^, CD44^*−*/lo^, CD25^*−*^) and DP stage^26^. For this reason, we FACS-sorted from P14 *Ada*^+*/*+^ and *Ada*^*−/−*^ thymi both Lin^neg^, CD25^+^ (DN2 + DN3) and CD4^+^/CD8^+^ DP populations and used high-throughput DNA sequencing (HTS) and FASTA sequence alignments^27^ to determine the splice junctions of the V(D)J regions and the nucleotide composition of N regions in the complementarity determining region 3 (CDR3) of TCRβ chain (Supplementary Fig. 5C-F left panels). More than forty different sequences were analyzed from each sample. Analysis of length distribution of the TCRβ chain CDR3 regions did not reveal significant differences between *Ada*^*−/−*^ and control immature CD25^+^ and DP thymocytes (Supplementary Fig. 5C,F middle panel). The length of the N regions varied with no significant differences between control and *Ada*^*−/−*^ cells, from 0 to 12 and from 0 to 15 nucleotides, with an average insertion size of 2.5 and 2.8 bp respectively in N1 and N2 regions (Supplementary Fig. 5G). Surprisingly, analysis of the N-region nucleotide compositions revealed no significant increase in the A-T content of the VD and DJ junctions of *Ada*^*−/−*^ cells, and similar nucleotide ratio (G + C/A + T) in both left and right N regions (Supplementary Fig. 5C-F right panels). We also found normal frequency distribution of V and J genes usage in *Ada*^*−/−*^ thymocytes compared to control cells (Supplementary Fig. 6A,B). We, therefore, did not detect an effect of the nucleotide pool imbalance on V(D)J recombination of immature thymocytes.

Finally, we evaluated if ADA deficiency could affect the signaling downstream the pre-TCRβ and abrogate the activation of specific thymic populations. In particular, we evaluated the expression of the activation/cell death marker CD69^28^ in DN3 and DN4, in resting conditions or after stimulation with immobilised anti-CD3e Ab. The proportion of cells expressing CD69 was similar in both *Ada*^*−/−*^ and *Ada*^+*/−*^ DN3 and DN4 thymocytes, in both the conditions (Supplementary Fig. 6C,D).

### dATP accumulation triggers apoptosis in Ada^−/−^ Lin^neg^, CD25^+^ (DN2 + DN3) thymocytes

**Figure 3:**
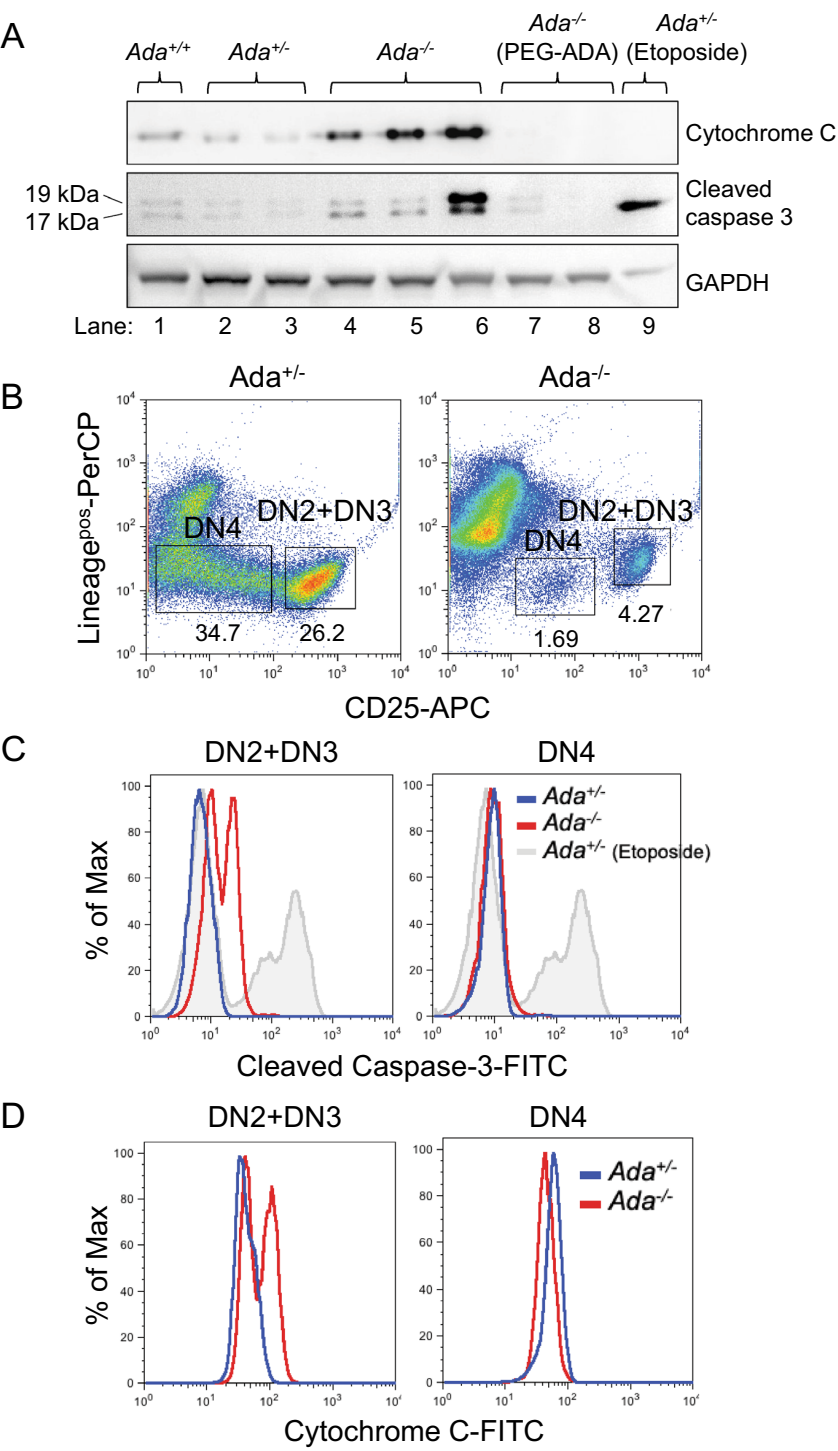
Immature thymocytes from ADA-deficient mice show increased expression of apoptotic markers. (**A**) Western blot analysis of protein lysates of thymi from P14 untreated mice (lane 1–6) and 3-month-PEG-ADA-treated mice (lane 7–8). As positive control for cleaved caspase-3 staining, unfractionated thymocytes were stimulated in vitro with the apoptosis-inducer etoposide (line 9). Anti-GAPDH stain was used as protein loading control. Full-length blots are presented in Supplementary Fig. 11. (**B**) FACS analysis of MACS-enriched Lin^neg^ thymocytes from untreated mice at P14. DN4 (Lin^neg^, CD25^*−*^) and DN2 + DN3 (Lin^neg^, CD25^+^) populations are gated. (**C**) Intracellular staining for cleaved caspase-3 of cell populations shown in (B). Unfractionated Lin^neg^ thymocytes were stimulated in vitro with etoposide as apoptosis positive control. (**D**) Intracellular staining for cytochrome C of cell populations shown in (B). FACS plots in (C) and (D) are representative of two replicate experiments (n = 4, 2).

Thymocyte development was arrested at the DN3 (Lin^neg^, CD44^*−*/lo^, CD25^+^) stage in *Ada*^*−/−*^ mice, thus preventing the DN3-to-DN4 (Lin^neg^, CD44^*−*/lo^, CD25^*−*^) transition and the β-selection. Having excluded any possible defect during the V(D)J recombination and in the ability of TSCs to support T cell development, we investigated apoptosis as a possible cause for the reduced transition from DN3 to DN4^4,29^. It has been shown that accumulated dATP causes release of cytochrome C from mitochondria, thus leading to the formation of an apoptosome and initiation of an apoptotic cascade which terminates with the cleavage of procaspase 3 into active caspase 3^30–33^. For this reason, we measured expression of cytochrome C and cleaved caspase 3 in protein lysates from controls and *Ada*^*−/−*^ thymi and found that both apoptotic markers were overexpressed in the ADA-deficient samples (Fig. 3A). Accordingly, we found overexpression of cytochrome C and cleaved caspase 3 in FACS sorted *Ada*^*−/−*^ Lin^neg^ CD25^+^ cells (DN2 + DN3) compared to control cells (Fig. 3B-D). Importantly, the small number of DN4 cells do not overexpress the apoptotic markers, indicating that some *Ada*^*−/−*^ DN3 cells manage to escape apoptosis and undergo pre-TCR-induced differentiation to DN4 and DP (Figs. 1C, 3C,D).

Altogether, our data suggests that dATP-induced apoptosis of the DN3 population may be responsible for the reduction in DN4 and DP cells and thymus atrophy in the *Ada*^*−/−*^ thymus.

### Long-term PEG-ADA treatment does not restore normal thymocyte number in Ada^−/−^ thymus

**Figure 4:**
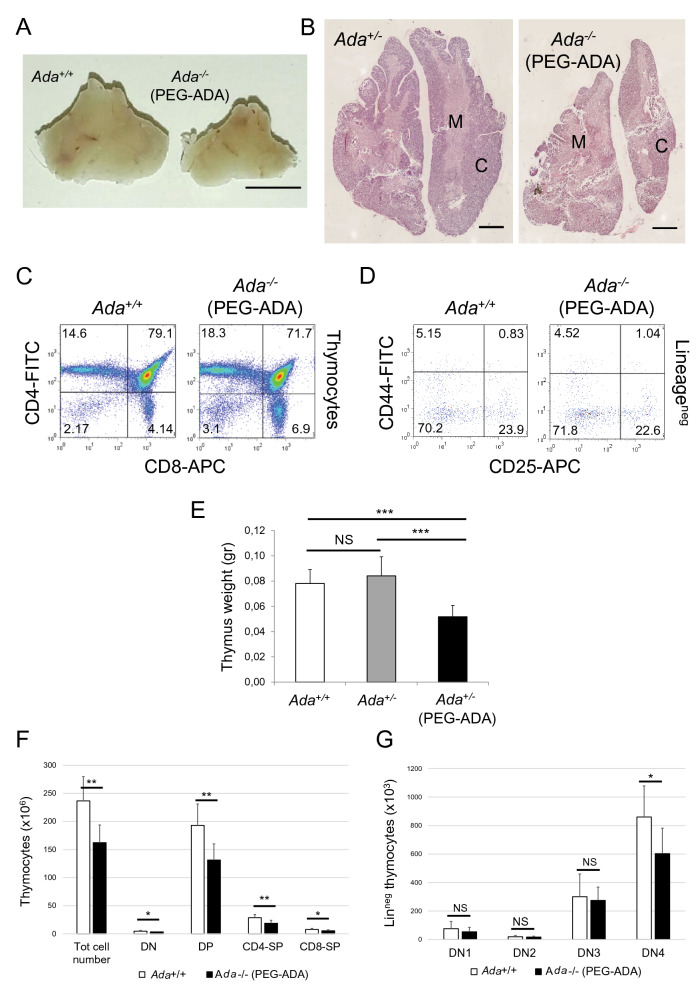
Thymus cellularity is not fully restored in PEG-ADA-treated mice. (**A**) In vivo images of adult thymi taken from control (*Ada*^+*/*+^) and 3-month-PEG-ADA-treated (*Ada*^*−/−*^) mice. (**B**) H&E staining of paraffin-embedded thymus sections from control (*Ada*^+*/*+^) and 3-month-PEG-ADA-treated (*Ada*^*−/−*^) mice. C = cortex, heavily filled with developing immature thymocytes; M = medulla, contains a mixture of helper (CD4 +) and cytotoxic (CD8 +) T cells. (**C** and **D**) FACS analysis of unfractionated thymocytes gated for: DN (CD4^−^, CD8^−^), DP (CD4^+^, CD8^+^), CD4-SP (CD4^+^, CD8^−^) and CD8-SP (CD4^−^, CD8^+^) populations (**C**), and Lin^neg^ thymocytes gated for: DN1 (CD44^+^, CD25^−^) DN2 (CD44^+^, CD25^+^) DN3 (CD44^−^, CD25^+^) DN4 (CD44^−^, CD25^−^) populations (**D**). All thymocytes were from control (*Ada*^+*/*+^) and 3-month-PEG-ADA-treated (*Ada*^*−/−*^) mice. (**E**) Bar graph representing weight of thymi from control (*Ada*^+*/*+^ and *Ada*^+/−^) and 3-month-PEG-ADA-treated (*Ada*^*−/−*^) mice (n = 5, 7, 11). (**F** and **G**) Bar graphs representing absolute cell numbers of populations shown in (C) and (D) (n = 6, 7). *P < 0.05, **P < 0.01 and ***P < 0.001. NS = statistically not significant.

It has been shown that maintaining high ADA activity in ADA-SCID patients’ plasma by weekly injections of PEG-ADA (Adagen®) eliminates systemically Ado and dAdo derived from nucleotide and nucleic acid turnover. This treatment protects lymphoid cells from apoptosis triggered by dAdo-induced dATP pool expansion and from other mechanisms, thus restoring protective immune function in most patients within approximately 2 to 4 months^11^. However, most patients remain T lymphopenic with low thymic output and peripheral T cell apoptosis^14,15,34^. This suggests that PEG-ADA treatment does not fully restore thymopoiesis. In order to find a possible explanation for this, we administrated PEG-ADA (1000 U/Kg body weight) to *Ada*^*−/−*^ mice and monitored immune recovery^10^. Injections were started between P7 and P10 and were given every 7 days for at least 3 months. As expected, ERT rescued ADA-deficient animals from the lethal phenotype and resulted in healthy fertile mice with a thymus with no sign of atrophy (Fig. 4A). Histological analysis revealed well-organized cytoarchitecture with a well-defined boundary between cortex and medulla (Fig. 4B). FACS analysis of thymocytes showed resolution of the developmental block at the DN3 stage (Fig. 4C,D) and normal distribution of both mature and immature thymocytes in the *Ada*^*−/−*^ thymus (Supplementary Fig. 7A,B). Western blot analysis of protein lysates from ERT thymi did not show overexpression of apoptotic markers such as cytochrome C and cleaved caspase 3 (Fig. 3A). However, although thymopoiesis appeared grossly normal in *Ada*^*−/−*^ thymi from ERT mice, we found a significant reduction in thymus weight compared to controls (Fig. 4E) and a significant reduction of about 50% in thymocyte number and the number of each thymocyte subpopulation in ERT mice (Fig. 4F). Interestingly and unlike blood from long-term ERT patients, we found circulating T and B lymphocytes (Supplementary Fig. 2C) and normalized cell counts (Supplementary Fig. 2D) in blood from PEG-ADA-treated mice at each time point analyzed (3, 6 and 9 months). The number of cells in the DN1, DN2 and DN3 populations was not different between ERT *Ada*^*−*/*−*^ and control mice, whereas the DN4 subset was significantly different (Fig. 4G).

HSPC numbers in the BM of ERT *Ada*^*−/−*^ mice were not significantly different from control (Supplementary Fig. 7C,D). These data suggest that ERT successfully normalizes the numbers of circulating lymphocytes and T cell progenitors which can reach and enter the thymus. Once in the thymus, these progenitors are also able to differentiate normally until the DN3 stage.

Altogether, our results suggest that PEG-ADA contributes to the resolution of dATP-induced apoptosis in the DN3 population. However, other mechanisms exist to impair differentiation beyond the DN4 stage, which are not repaired by PEG-ADA, thus contributing to the reduced thymus cellularity of ERT mice.

### Long-term PEG-ADA treatment does not restore normal splenocyte number in Ada^−/−^ spleen

*Ada*^*−/−*^ spleen after 3 months of PEG-ADA treatment showed, like *Ada*^*−/−*^ thymus, a striking increase in the number of splenocytes when compared to the untreated *Ada*^*−/−*^ spleens (Supplementary Fig. 3). However, we also noticed that cellularity and total number of CD4^+^ and CD8^+^ T-splenocytes in the *Ada*^*−/−*^ spleen were still reduced on PEG-ADA treatment compared to controls (Supplementary Fig. 3D,E). Accordingly, we found by tandem MS measurements that Ado and dAdo levels remained significantly high in treated spleens (Supplementary Fig. 3F), suggesting that ERT does not fully detoxify the splenic environment.

### ERT does not reduce Ado levels in Ada^−/−^ thymocytes

**Figure 5:**
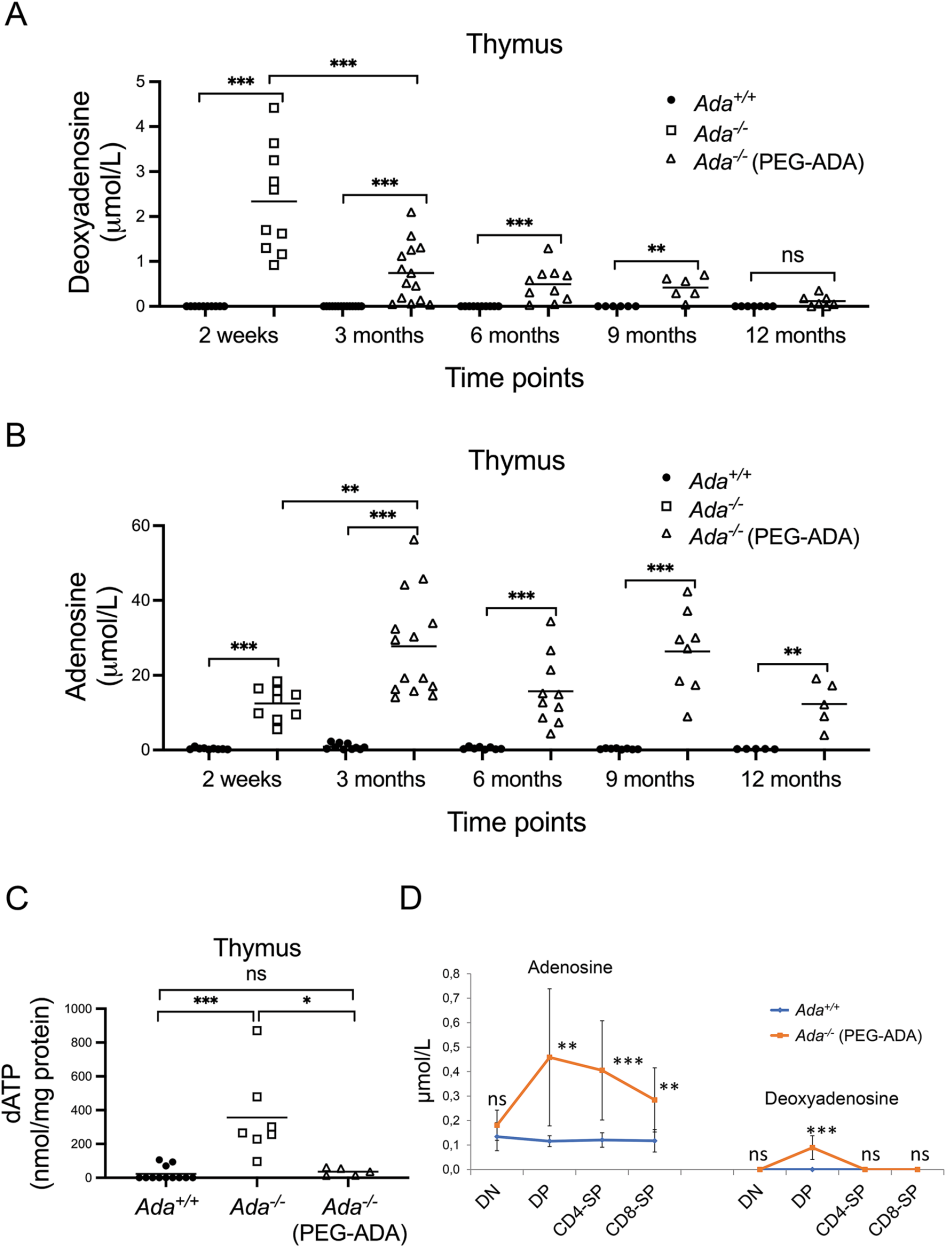
Incomplete detoxification of ADA metabolites in thymocytes from PEG-ADA-treated mice. Tandem MS measurement of dAdo (**A**) (2 weeks, n = 10, 10; 3 month, n = 14, 14; 6 months, n = 10, 10; 9 months, n = 5, 5; 12 months, n = 6, 6) and Ado (**B**) (2 weeks, n = 9, 9; 3 month, n = 10, 14; 6 months, n = 8, 10; 9 months, n = 8, 8; 12 months, n = 5, 5) levels in unfractionated thymocyte populations from control (*Ada*^+*/*+^), untreated and PEG-ADA-treated *Ada*^*−/−*^ mice at different time points. (**C**) Reversed phase HPLC measurement of dATP levels of unfractionated thymocyte populations from control (*Ada*^+*/*+^), untreated (2 weeks) and PEG-ADA-treated *Ada*^*−/−*^ (6 months) mice (n = 12, 7, 5). Plots in (A-C) were generated by GraphPad Prism 7 software. (**D**) Tandem MS measurement of Ado and dAdo levels in FACS-sorted thymocyte populations from control (*Ada*^+*/*+^) and PEG-ADA-treated *Ada*^*−/−*^ mice at 6-month-time point (n = 6, 8). Bars represent mean values. *P < 0.05, **P < 0.01 and ***P < 0.001. NS = statistically not significant.

The fact that *Ada*^*−/−*^ thymocyte and splenocyte numbers remained low compared to controls and were only partially detoxified from ADA metabolites after 3 months of ERT (Fig. 5A,B, Supplementary Fig. 3F) led us to conclude that a 3-month treatment with PEG-ADA is not sufficient for full immune recovery. For this reason, we treated *Ada*^*−/−*^ mice for 6, 9 and 12 months with PEG-ADA and monitored thymopoiesis at each time-point. Surprisingly, we found that thymus cellularity in ERT mice remained fixed at 50% of that of control thymi at all time points tested. We also evaluated cellularity after 2 weeks from the first injection and found the same significant reduction in cell number (Supplementary Fig. 7E). We therefore measured Ado, dAdo and dATP levels in both blood and thymus from ERT and control mice. Blood levels of ADA metabolites were normalized in ERT animals at each time point tested (Supplementary Fig. 8A-C). Likewise, dAdo levels in ERT thymi became normalized, even though the normalization process occurred gradually from 3-weeks to 12 months of treatment (Fig. 5A). Accordingly, dATP levels measured at the 6-month-time point were also comparable to those of control thymi (Fig. 5C). As expected, Ado levels remained relatively high in ERT thymi at all time points analyzed and never dropped below those of untreated mice (Fig. 5B).

We next FACS-sorted the four thymocyte subpopulations (DN, DP, CD4-SP and CD8-SP) from 6-month-ERT and control thymi to quantify by tandem MS the relative concentration of both Ado and dAdo (Fig. 5D). Ado levels in *Ada*^*−/−*^ DP, CD4-SP and CD8-SP cells were relatively high compared to dAdo levels which were instead normalized, whereas both Ado and dAdo levels in *Ada*^*−/−*^ DN cells were completely normalized. To test if partial detoxification was particularly related to thymus and spleen anatomy, we quantified ADA metabolite levels in non-hematopoietic organs such as lungs and kidney from ERT mice at 3-month-time point and found them normalized (Supplementary Fig. 8D).

These results clearly suggest that even though ERT provides an effective systemic detoxification in *Ada*^*−/−*^ mice leading to normalization of BM and stem/progenitor cell function, ERT does not reduce Ado levels in thymi from ERT treated mice.

### Ado accumulation does not interfere with thymocyte metabolism

**Figure 6:**
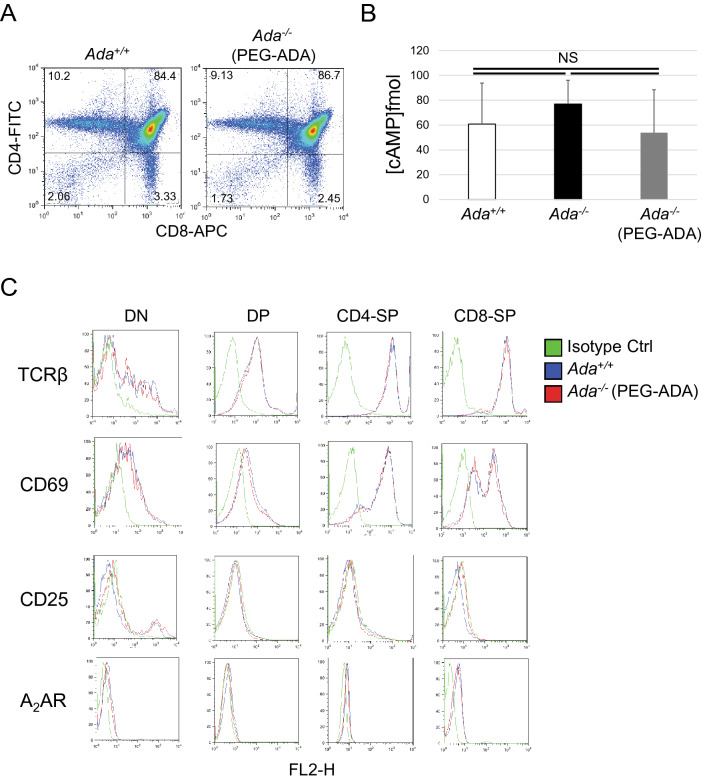
Ado accumulation does not interfere with thymocytes metabolism. (**A**) FACS plots of unfractionated thymocytes from control (*Ada*^+*/*+^) and 3-week-PEG-ADA-treated *Ada*^*−/−*^ mice and gated for DN (CD4^*−*^, CD8^*−*^), DP (CD4^+^, CD8^+^), CD4-SP (CD4^+^, CD8^*−*^) and CD8-SP (CD4^*−*^, CD8^+^) populations. (**B**) Bar graph representing cAMP levels of thymocytes shown in (A) and of thymocytes from untreated *Ada*^*−/−*^ mice at P14 (n = 3, 3, 3). (**C**) Each FACS-sorted cell populations shown in (A) were assessed for T cell surface receptors using PE-conjugated antibodies. FACS plots are representative of two replicate experiments (n = 3, 4). NS = statistically not significant.

Several studies have highlighted that Ado affects T cell development within the thymus^35^. It has been shown that Ado causes defective TCR signaling^4,36^, inhibits T cell activation and expansion by interfering with the progression of cell mitosis^37,38^ and triggers cAMP-induced thymocyte apoptosis through DNA cleavage^39–41^. In light of these studies, we attempted to find a link between intra- and extracellular accumulation of Ado and the reduced thymus cellularity in ERT mice. We investigated thymi from animals treated with PEG-ADA for only 3 weeks. We chose this time point because during the first few weeks of life thymi undergo intensive cell proliferation making them ideal for TCR signaling studies (Fig. 1A, Supplementary Fig. 7E). Surprisingly, we found that, although Ado and dAdo levels were not normalized in ERT thymi after only three PEG-ADA doses (Fig. 5A,B), the DN3 blockage was resolved (Fig. 6A) and both cAMP levels (Fig. 6B) and TCRβ, CD69, CD25 and Ado receptor A2AR profiles of freshly isolated thymocytes (Fig. 6C) were similar to controls.

To test a possible role of intracellular Ado and dAdo accumulation in T cell activation, we stimulated both thymocytes (Supplementary Fig. 9A,B) and T splenocytes (Supplementary Fig. 9C,D) from ERT and control mice with immobilised anti-CD3e Ab. FACS profiles of TCRβ, CD69, CD25 and A2AR receptors in ERT and control cells were comparable. In previous studies, Apasov et al. showed that extracellular Ado inhibits TCR-triggered upregulation of the activation marker CD69 on thymocytes in vitro, under conditions of inhibited ADA activity^42^. For this reason, we stimulated thymocytes from ERT and control mice with immobilised anti-CD3e Ab in presence of Ado or dAdo alone or in combination, and evaluated CD69 and TCRβ surface expression. Thymocytes from control mice were also cultured with a combination of the ADA inhibitor ENHA together with Ado or dAdo as positive control. As expected, CD69 expression on control CD4^+^ and CD8^+^ thymocytes was reduced under conditions of inhibited ADA activity (Supplementary Fig. 10A,C). Unexpectedly, CD69 and TCRβ expression were comparable between thymocytes from ERT and control mice even when cultured in presence of Ado and/or dAdo (Supplementary Fig. 10B,D,F).

**Figure 7:**
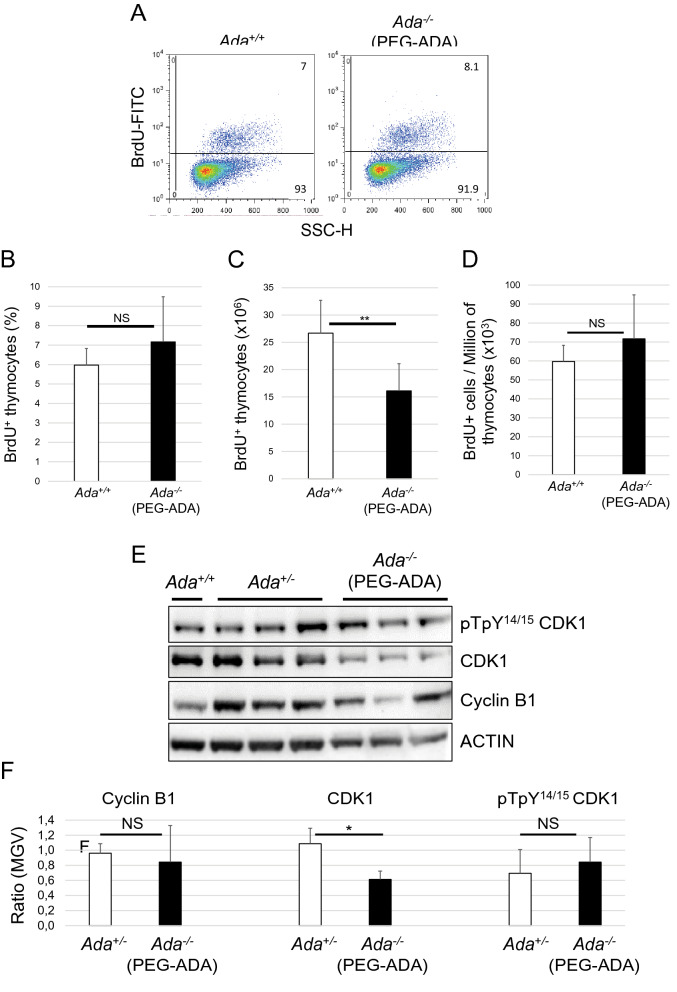
Ado accumulation does not interfere with thymocytes proliferation. (**A**) FACS plots of proliferating thymocytes (BrdU^+^) from control (*Ada*^+*/*+^) and 3-week-PEG-ADA-treated *Ada*^*−/−*^ mice. Bar graphs representing percentages (**B**), absolute (**C**) and relative (**D**) numbers of BrdU^+^ thymocytes shown in (A) (n = 6, 6). (**E**) Western blot analysis of protein lysates of thymocytes shown in (A). Anti-ACTIN stain was used as protein loading control. Full-length blots are presented in Supplementary Fig. 12. (**F**) ImageJ quantification of protein bands from Western blot film shown in (E). MGV = mean gray value. *P < 0.05, **P < 0.01 and ***P < 0.001. NS = statistically not significant.

Next, we investigated the proliferation ability of thymocytes in mice treated with PEG-ADA. Mice treated with PEG-ADA for 3 weeks were injected with BrdU, an analog of thymidine commonly used in the detection of proliferating cells in living tissues. Two hours after BrdU injection, thymi were dissected and thymocytes analyzed by FACS to detect the percentage of proliferating cells (BrdU^+^) (Fig. 7A). We found that, even though the absolute number of BrdU^+^ cells in ERT thymi was significantly reduced (Fig. 7C), the BrdU^+^ cell percentage value (Fig. 7B) and relative number (Fig. 7D) in ERT mice were comparable to those of control thymi. ERT thymi older than 3 months showed similar percentages of proliferating cells to those of control thymi. In order to corroborate this result, we also detected expression of cell-cycle-related proteins cyclin B1, CDK1 and pT-pY^14/15^CDK1 by Western blotting (Fig. 7E). Quantification of the blots revealed normal expression of cyclin B1 and normal CDK1 phosphorylation levels, even though expression of CDK1 resulted to be slightly underregulated (Fig. 7F).

## Discussion

Genetic defects in the purine salvage enzyme ADA lead to SCID with profound depletion of T, B and NK cell lineages both in humans and mice^2,43^.

Here, we have shown, for the first time in vivo, that production of mature T cells in ADA-deficient mice is profoundly inhibited by a developmental block of thymopoiesis at the CD44^*−*^CD25^+^ DN3 stage. This finding is consistent with data published by Thompson et al*.* concerning the abnormal accumulation of developing thymocytes in vitro in ADA-inhibited FTOCs^29^. Consequently, thymi in *Ada*^*−/−*^ mice develop severe postnatal atrophy characterized by decreased thymus weight and depletion of mature CD4^+^ and CD8^+^ thymocytes from the medulla, which in turn depletes the peripheral CD4^+^ and CD8^+^ T cell pools from blood and spleen. We also showed that the production of HSPCs by the BM in *Ada*^*−/−*^ mice was significantly reduced compared to controls. Therefore, we cannot exclude that the deleterious effect of the lack of ADA expression during development of HSPCs in *Ada*^*−/−*^ mice may contribute to thymus atrophy by affecting the production of thymic seeding progenitor populations.

It is worth noting that thymus cellularity at birth (P0) was in range with that of control thymi, suggesting that ADA expression is more essential during the postnatal than the fetal thymus development. This finding is in line with the evidence of the pivotal role of ADA during the early post-implantation embryonic development, where over 95% of fetal ADA is present in the placenta^44,45^. Additionally, we found that, unlike DP cells, the number of CD44^+^CD25^*−*^ DN1 and CD44^+^CD25^+^ DN2 cells in *Ada*^*−/−*^ thymi were comparable to that of controls, which reflects a more predominant role of ADA in DP cell turnover. Altogether, these findings demonstrate that ADA activity varies markedly during mouse ontogeny and suggests that fundamental differences in nucleotide metabolism may exist in different cell populations or subpopulations at different stages of development^46,47^.

We next investigated the potential mechanisms that may cause arrest and accumulation of the DN3 population in *Ada*^*−/−*^ mice. We excluded the possibility that accumulation of toxic metabolites may interfere with the development, maturation and function of thymic stroma cells (TSCs) because FTOCs using thymic lobes from *Ada*^*−/−*^ mice and wild-type BM-derived HSPCs did not show any impaired ability to support T cell development. However, FTOCs with thymic lobes from *Ada*^+*/*+^ mice and *Ada*^*−/−*^ BM-derived HSPCs showed reduced thymocyte viability and severely impaired T cell differentiation. These in vitro experiments suggested that the thymic development defects in *Ada*^*−/−*^ mice are due to a T cell progenitor-intrinsic abnormality rather than a failure of appropriate support from the thymic stromal epithelium.

We did not find any evidence of alterations in TCR signaling or TCRβ chain V(D)J recombination that may impede the DN3 to DN4 transition or interfere with the β selection. Likewise, the CD69 expression profile, which is altered in pre-TCR/CD3 signaling-deficient mice^28^, was found to be similar in *Ada*^*−/−*^ and control DN thymocytes. Analysis of the nucleotide composition in the V(D)J junctions showed no significant difference in the GC/AT ratios of the N-regions. The frequency distribution of V and J genes usage and expression of TCRβ in *Ada*^*−/−*^ immature thymocytes were also found normal. These data clearly suggest that the nucleotide pool imbalance in *Ada*^*−/−*^ mice has no effect on the V(D)J recombination frequency. This is in contrast with data published by Gangi-Patterson et al*.* which associated B-cell dysfunction to reduced V(D)J recombination frequency and altered N-region composition as a consequence of increased dATP levels in ADA-SCID patients^6^.

Finally, we investigated the relationship between intracellular dATP accumulation and dATP-induced apoptosis levels in *Ada*^*−/−*^ thymocytes^30–33^. We quantified expression of cytochrome C and cleaved caspase 3 death markers in *Ada*^*−/−*^ thymocytes and found that DN3 cells were particularly sensitive and more prone to develop apoptosis, suggesting that dATP-induced apoptosis may be responsible for the thymus atrophy.

ERT with twice-a-week PEG-ADA administration for the treatment of ADA-SCID has been successfully used since 1986^48^. However, in the long-term, patients undergoing ERT have shown variable and incomplete immune reconstitution^12,14,15^, despite plasma ADA activity being stable and higher than the physiologic levels at all times^49^. Interestingly and unlike ERT, BM-transplanted and gene therapy-treated patients have shown long-term immunological recovery and stable metabolic correction^13,50,51^. The incomplete immune recovery in ERT patients remain so far without any formal explanation. It may be associated with the general clinical condition of the patient, their age or the level of residual thymic activity at the time of PEG-ADA treatment is started. Some patients also develop neutralizing anti-ADA antibodies which reduce activity of circulating PEG-ADA^52^. Here, we monitored for 12 months the immune reconstitution of ADA-deficient mice receiving weekly doses of PEG-ADA. We found in treated mice for at least 3 months a normal thymus cytoarchitecture with a well-defined boundary between cortex and medulla, an increased thymus weight reflecting increased thymocyte number and maturation, production and release of mature T lymphocytes in blood and spleen and normalized BM-derived lymphoid progenitor numbers. We also confirmed that the immunologic improvement in the thymus of *Ada*^*−/−*^ mice is mainly due to resolution of apoptosis, as previously shown^4^. However, our study demonstrated for the first time that the specific resolution of apoptosis in the DN3 cells is crucial for the reconstitution of an efficient thymopoiesis. Additionally and unlike patients^14,15^, we found normalized and stable peripheral blood lymphocyte counts suggesting an efficient thymic output in ERT-treated mice, even though thymus and spleen cellularity remained significantly reduced compared to controls.

We next reasoned that partial detoxification of treated ADA-deficient mice from toxic metabolites may be the cause of the subnormal cellularity in thymus and spleen. Levels of Ado and dAdo in thymus and spleen were indeed found relatively elevated throughout, even though dAdo levels progressively decreased at each time point tested. Consequently, dATP levels were lowered enough to prevent the dAdo/dATP-induced thymocyte apoptosis. As expected, however, blood samples from ERT mice resulted to be fully detoxified from all ADA metabolites. Whether higher doses and/or more frequent administration of PEG-ADA might provide improved detoxification in lymphoid organs and better immune recovery in mice remains to be determined. However, we believe that the extremely high cost of the medication and the limited volumes that can be administered in mice, make dose escalation impractical.

It is well known that adenosine acts as an extracellular signaling molecule via activation of G-protein-coupled receptors^8,53^ and regulates various biological functions in the thymus^4,35–38^, including cAMP-induced thymocyte apoptosis^41,54^. However, we were not able to correlate the reduced thymus cellularity in PEG-ADA-treated mice to the persistent high concentration of Ado. cAMP levels, thymocytes proliferation rates, expression profiles of maturation (CD3e, TCRβ), activation (CD69) and proliferation (CD25) markers, expression of adenosine receptor A2AR and cell-cycle-related proteins were all not found to be significantly different from control values. Altogether, these results suggest that a new and unexplored role for adenosine in controlling thymopoiesis may account for the reduced cellularity in ERT mice.

In conclusion, we have shown that while circulating PEG-ADA acts as a metabolic sink detoxifying systemic levels of ADA metabolites in *Ada*^*−/−*^ mice leading to normalization of blood cell counts and BM stem/progenitor cell function, ERT does not reduce the intra-/extracellular levels of Ado in susceptible lymphoid organs. We cannot exclude that specific anatomical peculiarities of thymus and spleen may prevent or slow down the diffusion of metabolites with the concentration gradient to the plasma. Our findings have also strong implications for understanding the incomplete reconstitution of lymphocyte pools in patients treated with long-term ERT and confirm an underlying limitation of ERT therapy, highlighting the need to further evaluate alternative interventions such as allogeneic HSPC transplantation or autologous HSPC gene therapy.

## Methods

### Animals

ADA-deficient mice^9^ (FVB; 129-Adatm1Mw Tg(PLADA)4118Rkmb/J) were purchased from the Jackson Laboratory (Bar Harbor, ME) and bred at the UCL GOS Institute of Child Health’s animal barrier facility under specific pathogen-free conditions. All animals were kept in a 12 h day-night cycle environment with controlled temperature and humidity, and everyday supply of sterile food and water. Newborns were routinely genotyped by standard PCR amplification of DNA from toe biopsies (Supplementary Fig. 1G).

Heterozygous (*Ada*^+*/−*^) mice are healthy, fertile and phenotypically indistinguishable from wild-type (*Ada*^+*/*+^) mice. Therefore, both lines were used as control for the experiments. Homozygous (*Ada*^*−/−*^) mice were either sacrificed at P14, before development of any severe SCID-like phenotype^9^, or maintained under ERT for 3, 6, 9 and 12 months by weekly intraperitoneal injections of PEG-ADA (Adagen®, 1000 U/kg of body weight)^10^. Injections were started between P7 and P10 and regularly continued until animals were sacrificed.

### Blood test

Blood samples were collected in BD Microtainer MAP K2EDTA (363,706, BD Diagnostics) and total lymphocyte numbers quantified by XE-5000 Automated Hematology System (Sismex).

### FACS analysis and antibodies

Multi-color FACS experiments were performed using FACSCalibur and LSR-II (BD Biosciences) and data analyzed by either BD CellQuest Pro Version 6.0 (https://ncxt.lbl.gov/files/lab/cellquestprouserguide.pdf) or BD FACSDiva Version 8.0.1 (https://www.bu.edu/flow-cytometry/files/2010/10/BDFACSDivaSoftwareQuickStart.pdf) software. FACS plots were then generated by BD FlowJo v10 software (FlowJo LLC, USA) (http://www.flowlab-childrens-harvard.com/yahoo_site_admin/assets/docs/FLOWJO_BASIC_TUTORIAL.29461821.pdf).

Thymocytes and mature T and B cells from blood and spleen were analyzed for surface receptor expression with anti-CD4-FITC (553729), anti-CD4-PE (553730), anti-CD8a-APC (553035), anti-CD45R/B220-PerCP (561086) and anti-CD3-PE (555275) from BD Pharmingen, anti-TCRβ-PE (130-104-859), anti-CD25-PE (130-102-788) and anti-CD69-PE (130–115-575) from Miltenyi Biotec and anti-A2AR-PE (www.novusbio.com/NBP1-39474PE). T cells tested for surface receptor expression were either left untreated or stimulated overnight at 37 °C with immobilized anti-CD3e Ab. A 24-well plate was coated with 2 µg/mL anti-mouse CD3 (17A2) Ab (100201, BioLegend) for 1 h at 37 °C then washed and covered with ~ 1 × 10^6^ cells per well for stimulation. Cells were then harvested and stained with appropriate antibodies.

The effects of Ado and dAdo stimulation on surface receptor expression were analyzed by FACS after incubating thymocytes as previously reported^42^. A 96-well plate was first coated overnight at 4ºC with 20 µg/mL CD3e Ab, washed and then ~ 2 × 10^5^ cells per well were plated for stimulation. Adenosine (A4036, Sigma-Aldrich) and 2′-deoxyadenosine (D7400, Sigma-Aldrich) were added at the culture medium either alone or in combination at the final concentration of 100 μM each, with or without 10 μM ADA inhibitor EHNA hydrochloride (E114, Sigma-Aldrich).

The Lin^neg^ cell populations from thymi were analyzed by FACS with removal of mature cells using the following cocktail of biotinylated anti-mouse antibodies (Miltenyi Biotec): anti-CD45R (B220) (130-101-998), anti-CD19 (130-101-951), anti-TER119 (130-102-016), anti-NK1.1 (130-102-037), anti-CD11b (130-098-582), anti-CD8α (130-102-023), anti-CD3e (130-101-990), anti-TCRβ (130-104-855) , anti-TCRγδ (130-102-118) and anti-CD11c (130-101-999) and gated out with anti-biotin-PerCP (130-098-799). Lin^neg^ cells were then further gated with anti-CD44-FITC (130-102-933) and anti-CD25-APC (130-102-787) (Miltenyi Biotec) to discriminate the four DN populations: DN1 (CD44^+^, CD25^*−*^), DN2 (CD44^+^, CD25^+^), DN3 (CD44^*−*/lo^, CD25^+^) and DN4 (CD44^*−*/lo^, CD25^*−*^)^19,20^.

The Lin^neg^ thymocytes used in apoptosis assays were firstly enriched by negative selection through MACS separation columns (Miltenyi Biotec) after staining the whole thymocyte population with anti-mouse CD4 (130-049-201) and CD8a (130-049-401) coupled magnetic MicroBeads (Miltenyi Biotec). The throughflow was then stained for FACS analysis with the cocktail of biotinylated antibodies described above (including anti-CD4, 130-109-412, Miltenyi Biotec) to gate out the lineage positive cells with anti-biotin-PerCP and to discriminate DN4 (Lin^neg^, CD25^*−*/low^) from DN2 + DN3 (Lin^neg^, CD25^+/hi^) population with anti-CD25-APC. The MACS-sorted cells were also intracellularly stained for apoptotic markers with the following FITC-conjugated antibodies: monoclonal cytochrome C (6H2) (11-6601-82, Invitrogen) and anti-cleaved caspase-3 (Asp175) (#9669 Cell Signaling technology). As positive control for the apoptosis assays by FACS and Western blot, thymocytes were treated with the apoptosis inducer etoposide (25 μM, ab120227, abcam) for 5 hours^55^.

The MACS-sorted Lin^neg^ thymocytes used for evaluating the expression of intracellular TCRβ (iTCRβ), surface TCRβ and CD69 were FACS-enriched as described above but excluding anti-CD3e and anti-TCRβ from the cocktail of biotinylated antibodies. Lin^neg^ cells were then further gated with anti-CD44-FITC and anti-CD25-APC to discriminate the four DN populations. The (i)TCRβ and CD69 cellular expression was estimated with anti-TCRβ-PE and anti-CD69-PE. Cells tested for CD69 expression were either left untreated or stimulated overnight at 37 °C with immobilized anti-CD3e Ab (10 µg/mL) as described above.

The BM Lin^neg^ cells were firstly magnetically-sorted using the Lineage Cell Depletion kit (Miltenyi Biotec, 130-090-858) and then stained and gated for SCA1-FITC (130-102-831) and CD117/cKIT-APC (Miltenyi Biotec, 130-102-796).

### Western blot and antibodies

Protein samples were prepared in lysis buffer containing 1% Brij-97, resolved by SDS-PAGE, transferred to PVDF membranes (Millipore) and then immunoblotted with different antibodies: polyclonal sheep anti-human/mouse ADA Ab (AF7048, R&D Systems), polyclonal rabbit anti-cleaved caspase-3 (Asp175) Ab (#9661, Cell Signaling technology), monoclonal mouse anti-cytochrome C [7H8.2C12] Ab (ab13575, abcam), monoclonal mouse anti-CDK1 (A17) Ab (33-1800, Thermo Fisher Scientific), polyclonal goat anti-human/mouse cyclin B1 Ab (AF6000, R&D System) and polyclonal rabbit anti-phospho-CDK1 (Thr14, Tyr15) Ab (44-686G, Thermo Fisher Scientific). Protein loads were checked by using either monoclonal anti-GAPDH (14C10) Rabbit mAb (#2118, Cell Signaling technology) or polyclonal rabbit anti-ACTIN (A2066, Sigma-Aldrich). Quantification of the protein bands was performed by ImageJ software (http://www.yorku.ca/yisheng/Internal/Protocols/ImageJ.pdf).

### Fetal thymus organ culture (FTOC)

Technical details were previously described^24^. Briefly, E15 thymic lobes were firstly treated with dGuo for 5–7 days and then overnight cocultured in a hanging drop with mouse BM Lin^neg^ cells magnetically-sorted (130-090-858 Miltenyi Biotec). Seeded lobes were then cultured for 3 weeks on 0.8 μm isopore membrane filters (Millipore) supported by cotton gauze. Developing thymocytes were then analyzed by FACS with anti-mouse CD4-FITC and CD8-APC.

### Proliferation assays

For in vivo labelling of thymocytes, BrdU (1 and 2 mg for younger and adult mice, respectively) was intraperitoneally injected according to manufacturer's protocol (559619, BD Pharmingen BrdU Flow Kit). Two hours after injection, cells were isolated from thymi, permeabilized with BD Cytofix/Cytoperm buffer and stained with FITC-labeled anti-BrdU monoclonal, CD4-PE and CD8-APC antibodies, and analyzed by FACS.

### Histological analysis of the thymus

Thymi were fixed in 10% formalin and embedded in paraffin according to standard protocols. Eight μm-thick tissue sections were then stained with H&E and mounted for histopathological analysis by optical microscope (Zeiss Axioplan).

### Nucleotide quantification

The Ado and dAdo quantitative analysis was performed by tandem MS as described elsewhere^56^. Briefly, thymocyte suspensions and blood (~ 50 μL) were dropped on Whatman 903 cards (WHA10531018, Merck) and shipped to the MS lab at room temperature. Cards were stored at − 80 °C, in sealed plastic bags containing desiccant and humidity indicator card, until analysis. Samples were prepared by punching a 3.2 mm disk from each card and extracted by dispensing 300 µL of a mixture of methanol and water (2:1, v/v) containing ribose-1-^13^C-adenosine and ^13^C_5_ deoxyadenosine (10 µmol/L) as internal standard. Samples were then shaken on a vortex system for 25 min at 37 °C and then transferred to a 96-well plate. Three µL of each sample were injected into a Synergi fusion-RP column (150 mm × 2 mm i.d.; 4 µm particle size) coupled to a the Sciex API 4000 TQ mass spectrometer for the MS/MS experiments.

The dATP quantification was performed by reversed phase HPLC. Thymocytes and red blood cells (RBC) were homogenized with 400 μL of cold 10% TCA in a 1.5 ml Eppendorf tube. The tube was spun at 12,000 rpm in a microfuge for 2 min, and the supernatant removed to a clean tube. This was washed twice with water-saturated di-ethyl ether to remove the TCA and 1 μL was injected on a Water Acquity UPLC system with PDA detection. The nucleotides were separated by reverse phase, using a Waters BEH C18 1.7 μm (2.1 × 150 mm) column, with a 40 mM acetate buffer and a gradient to 10% methanol over 12 min, and a run time of 15 min. The nucleotides were identified by UV spectrum and retention time. The protein pellet from each sample was dissolved in 500 μL of 0.1 M NaOH and the protein measured by the Folin Lowry method, and the results expressed as nmol/mg protein.

The intracellular cAMP level of thymocytes was measured according to manufacturer’s instructions using Amersham cAMP Biotrak competitive Enzymeimmunoassay (EIA) System (GE Healthcare Life Sciences, RPN2251). 100 μL of sample cells (1 × 10^6^ cells/ml) were lysed and measured in 96-well microplates. Samples and cAMP standards (for non-acetylation assays in the range 25–6400 fmol/well) were assayed in duplicate.

Data from MS and HPLC measurements were analyzed and graphed by GraphPad Prism 7 (GraphPad Software Inc, USA), https://www.graphpad.com/scientific-software/prism/.

### VDJ recombination analysis via HTS

TCR β-chain sequencing was performed on wild-type DN and DP, and ADA-deficient DN and DP. Libraries were prepared as described before^27,57^. Briefly, cDNA was synthesized from total RNA samples, an oligonucleotide was then ligated on the V-region where two random hexamers were also introduced, followed by three steps of PCR where amplification and extension (to add indices and Illumina adaptors) of the ligation product was completed. After each amplification step, products were purified using Agencourt AMPure XP magnetic beads (BeckmanCoulter A63880) according to the manufacturer’s instructions. After pooling and denaturing the libraries, a final concentration of 12 pM was added in a MiSeq 500-V2 Illumina cartridge for sequencing on a MiSeq machine at UCL Genomics. The resulting FASTQ files were demultiplexed and error-corrected using the Decombinator (Version 3) analysis pipeline^27,58^. Briefly, sequences are separated by source sample, and the V gene, V deletion, insertion sequence, J deletion, and J gene are identified (resulting in clonotypes). Following UMI and sequencing error correction, files are produced giving the above information as well as the CDR3 amino acid sequence. All further analyses were performed using R (version 3.4.2). D gene beta chain sequences GGGACTGGGGGGGC (TRBD1*01) and GGGACAGGGGGC (TRBD2*01) were retrieved from IGMT/GENE-DB^59^. A core of eight central nucleotides (ACTGGGGG and GACAGGGG) were used to identify the D gene from VDJ recombination for the purposes of characterizing the sequences. Only sequences in which the whole TRBD1*01 or TRBD2*01 sequence could be identified and removed were used for analysis of N region length and composition in each sample.

### Statistics

All measurements were repeated at least three times independently. Data collected from 3 to 14 mice were then pooled and graphed. Age-matched animals were randomly used in each experiment. Means, SD and statistical comparison by two-tailed, paired or homoscedastic Student’s t-Test were performed using Microsoft Excel software. Statistical analysis by two-tailed Fisher's Exact test was made using GraphPad Prism 7 (GraphPad Software Inc, USA). *P* < 0.05 was considered significant for all tests.

### Animal study approval

All animals in this study were maintained in accordance with the UK Home Office regulations. All experiments were conducted after approval by the University College London Animal Welfare and Ethical Review Body (project license 70/8241). We also confirm that this study is reported in accordance with ARRIVE guidelines.

## Supplementary Information

Supplementary Information.
