# Author Correction: Metabolite and thymocyte development defects in ADA-SCID mice receiving enzyme replacement therapy

**DOI:** 10.1038/s41598-021-03903-7

**Published:** 2021-12-15

**Authors:** Federico A. Moretti, Giuliana Giardino, Teresa C. H. Attenborough, Athina Soragia Gkazi, Ben K. Margetts, Giancarlo la Marca, Lynette Fairbanks, Tessa Crompton, H. Bobby Gaspar

**Affiliations:** 1 UCL Great Ormond Street Institute of Child Health, London, UK; 2 Department of Experimental and Clinical Biomedical Sciences, University of Florence and Newborn Screening, Clinical Chemistry and Pharmacology Lab, Meyer Children’s Hospital, Florence, Italy; 3 Purine Research Laboratory, St Thomas’ Hospital, London, UK

Correction to: *Scientific Reports* 10.1038/s41598-021-02572-w, published online 01 December 2021

The Supplementary Information files published with this Article contained an error where the labels were incorrect. The Supplementary Information files have been combined into a single composite file.

These errors have now been corrected in the Supplementary Information file that accompanies the original Article.

