# Supplementary material for: Metabolite and thymocyte development defects in ADA-SCID mice receiving enzyme replacement therapy
Source: Sci Rep. 2021 Dec 1;11:23221. doi: 10.1038/s41598-021-02572-w (PMC8636570; doi:10.1038/s41598-021-02572-w)
Supplement: Supplementary file 1 — Supplementary Information. [file 41598_2021_2572_MOESM1_ESM.pdf]

SUPPLEMENTARY MATERIAL

Metabolite and thymocyte development defects in ADA-SCID mice receiving enzyme replacement therapy

Federico Andrea Moretti[1*], Giuliana Giardino[1], Teresa C. H. Attenborough[1], Athina Soragia Gkazi[1], Ben K. Margetts[1], Giancarlo la Marca[2], Lynette Fairbanks[3], Tessa Crompton[1] and H. Bobby Gaspar[1]

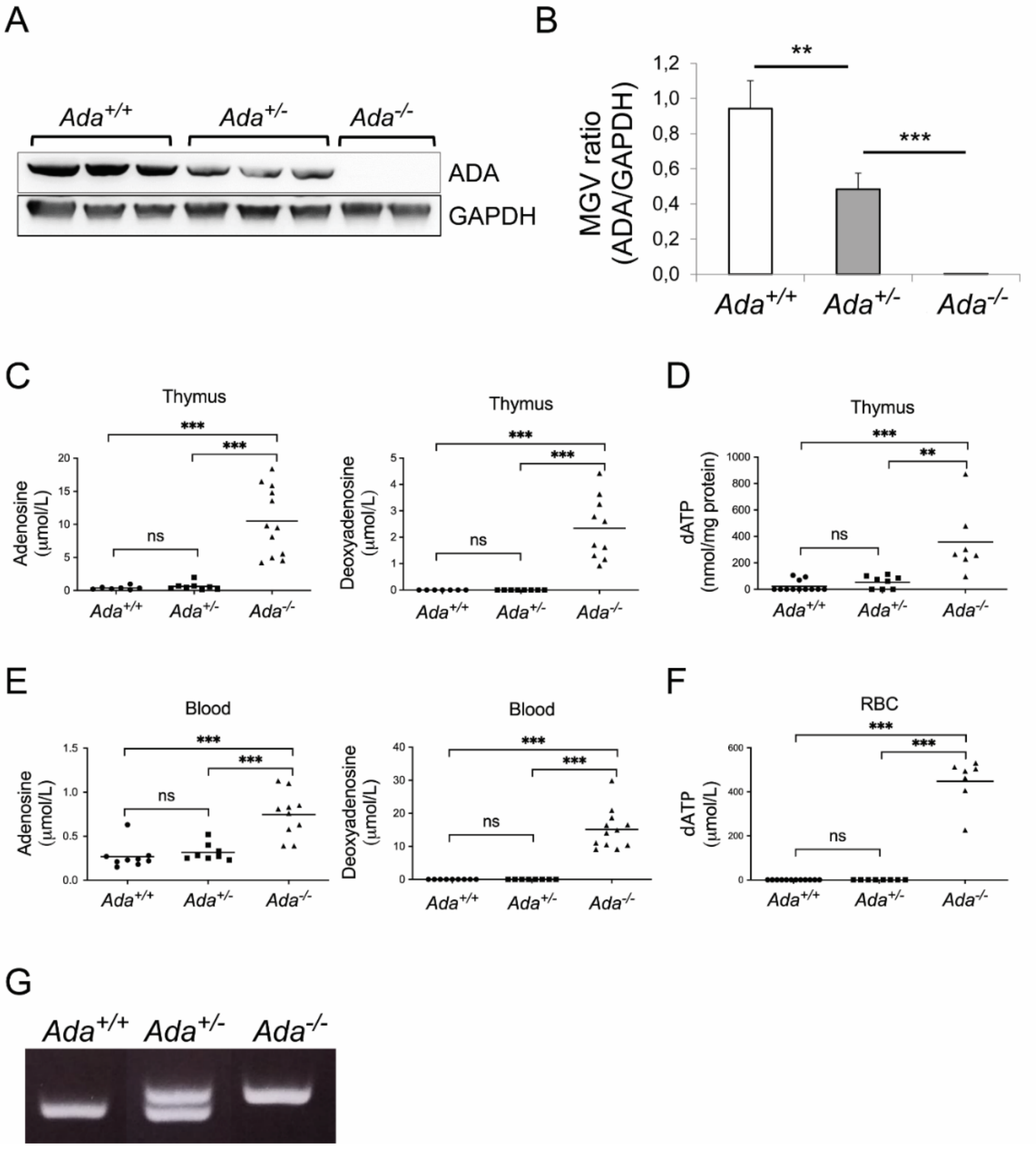

Figure S1. ADA deficiency is associated with accumulation of purine metabolites in tissues

(**A**) Western blot analysis of protein lysates of thymocytes from *Ada*$^{+/+}$, *Ada*$^{+/-}$ and *Ada*$^{-/-}$ mice showing the different expression of ADA enzyme. Anti-GAPDH stain was used as protein loading control. Full-

length blots are presented in Figure S13. (**B**) ImageJ quantification of protein bands from Western blot film shown in (A) (n= 3, 3, 2). Data represent means ± SD. (**C** and **D**) Tandem MS-measured Ado (n= 7, 8, 12) and dAdo (n= 7, 8, 10) (C) and reversed phase HPLC-measured dATP (n= 12, 8, 7) (D) levels in unfractionated thymocyte populations from control (*Ada*$^{+/+}$, *Ada*$^{+/-}$) and untreated *Ada*$^{-/-}$ mice at postnatal day 14 (P14). (**E** and **F**) Tandem MS-measured Ado (n= 9, 8, 10) and dAdo (n= 9, 8, 12) (F) and reversed phase HPLC-measured dATP (n= 12, 8, 7) (G) levels in blood from control (*Ada*$^{+/+}$, *Ada*$^{+/-}$) and untreated *Ada*$^{-/-}$ mice at P14. Plots in (C-F) were generated by GraphPad Prism 7 software. (**G**) Agarose gel showing PCR-amplified genomic DNA bands to identify the three ADA genotypes: WT (*Ada*$^{+/+}$), heterozygous (*Ada*$^{+/-}$) and knock-out (*Ada*$^{-/-}$) mice. Full-length gel is presented in Figure S14. RBC= red blood cells. MGV= mean gray value. Statistical analysis was performed using the two-tailed homoscedastic Student's t-Test. P values <0.05 were considered significant. Bars represent mean values. *P <0.05, **P <0.01 and ***P <0.001. NS= statistically not significant.

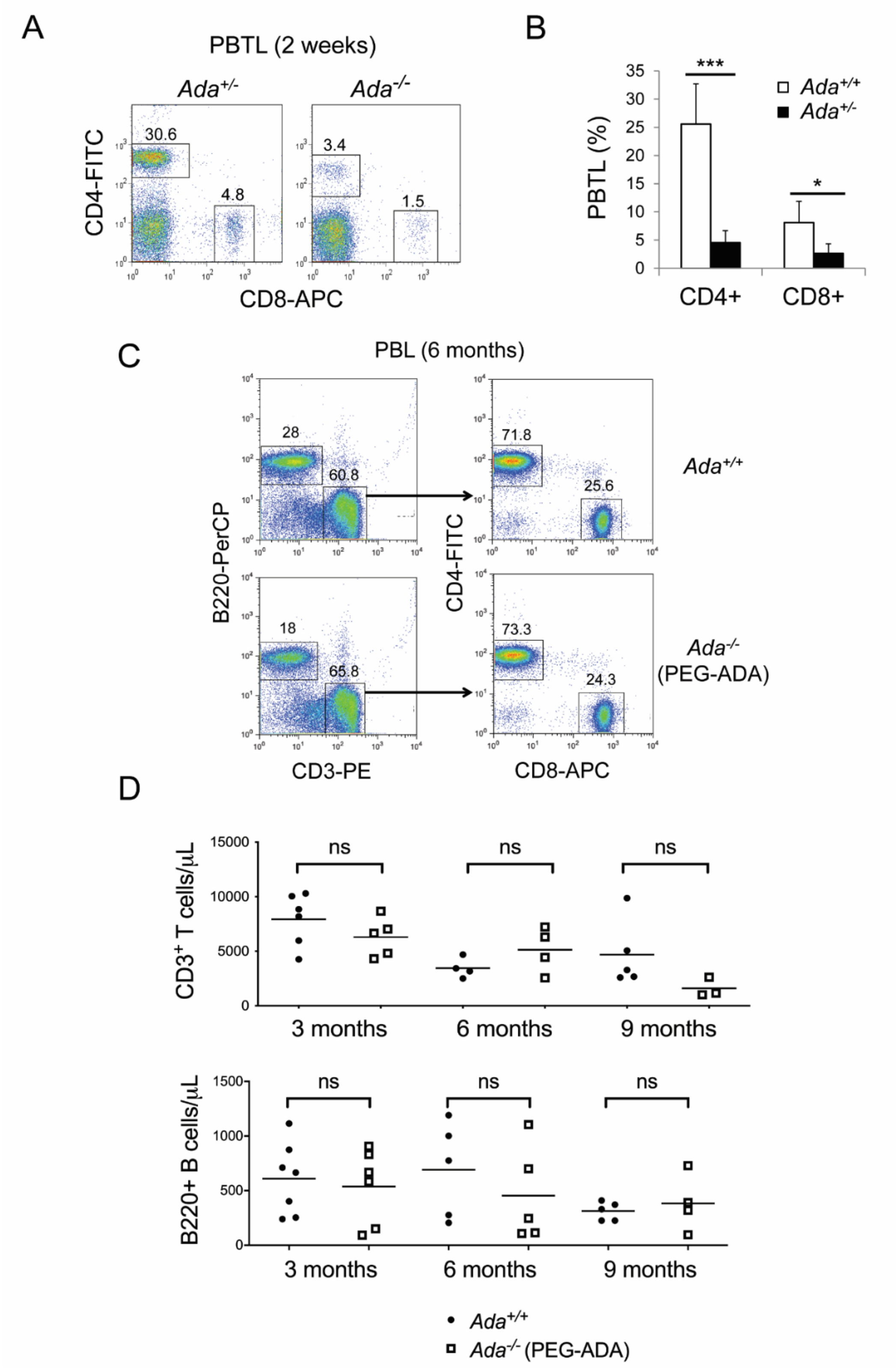

Figure S2. Enzyme replacement therapy normalizes peripheral blood lymphocyte counts in ADA-deficient mice

(**A**) FACS analysis of peripheral blood T lymphocytes (PBTL) from control (*Ada*$^{+/-}$) and untreated *Ada*$^{-/-}$ mice at P14. (**B**) Bar graph representing percentages of T-lymphocyte shown in (A) (n= 7, 5). (**C**) FACS

analysis of peripheral blood T ($CD3^+/CD4^+$ and $CD3^+/CD8^+$) and B ($B220^+$) lymphocytes (PBL) from control (*Ada*$^{+/+}$) and 6-month-PEG-ADA-treated (*Ada*$^{-/-}$) mice. (**D**) Peripheral blood T ($CD3^+$) and B ($B220^+$) lymphocyte counts in control (*Ada*$^{+/+}$) and PEG-ADA-treated *Ada*$^{-/-}$ mice at different time points (3 months, n= 7, 6; 6 months, n= 5, 5; 9 months, n= 5, 4). Plots in (D) were generated by GraphPad Prism 7 software. Quadrant numbers represent relative cell percentages. *P <0.05, **P <0.01 and ***P <0.001. NS= statistically not significant.

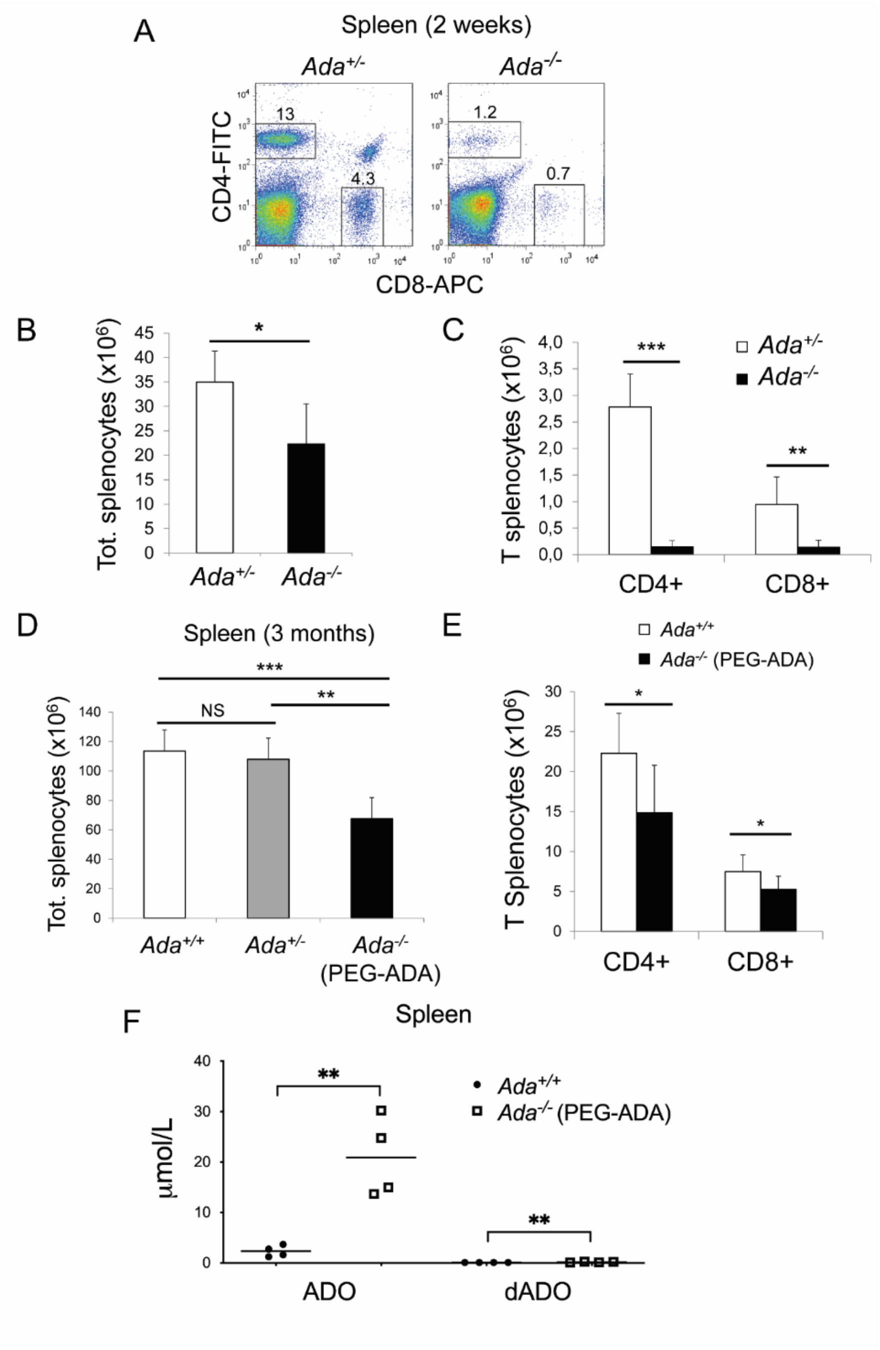

Figure S3. Enzyme replacement therapy does not normalize splenocyte numbers in ADA-deficient mice

(**A**) Percentage of T-splenocytes, (**B**) total number of cells and (**C**) absolute number of CD4$^+$- and CD8$^+$-T cells from spleens of control (*Ada*$^{+/-}$) and untreated *Ada*$^{-/-}$ mice at P14 (n= 6, 5). (**D**) Total number of cells, and (**E**) absolute number of CD4$^+$- and CD8$^+$-T cells from spleens of control (*Ada*$^{+/+}$, *Ada*$^{+/-}$) and 3-month-PEG-ADA-treated *Ada*$^{-/-}$ mice (n= 8, 3, 8). (**F**) Tandem MS-measured Ado and dAdo levels in unfractionated splenocytes from control (*Ada*$^{+/+}$) and 3-month-PEG-ADA-treated *Ada*$^{-/-}$ mice (n= 4, 4). Plot in (F) was generated by GraphPad Prism 7 software. *P <0.05, **P <0.01 and ***P <0.001. NS= statistically not significant.

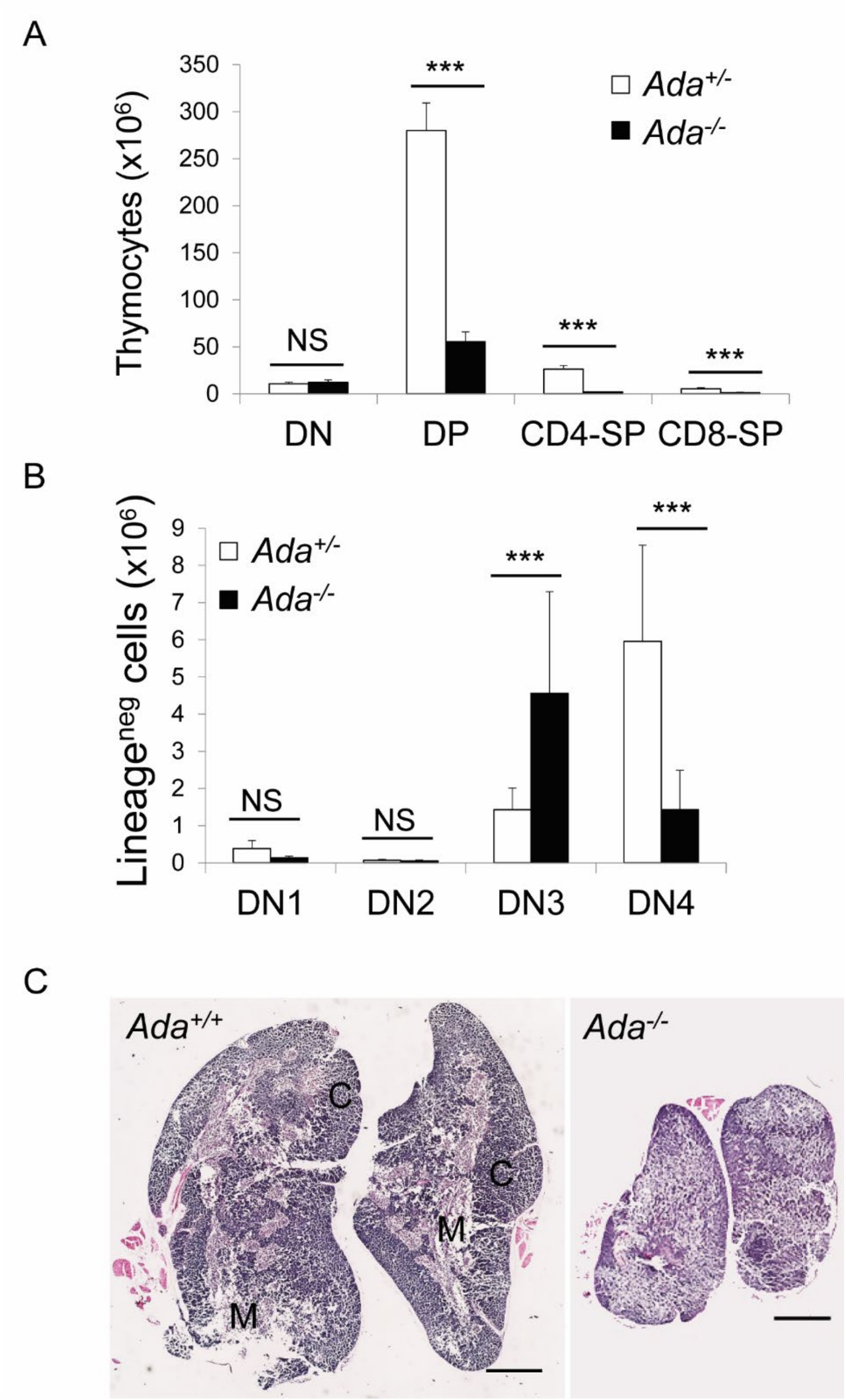

Figure S4. ADA-deficient mice show T cell development blockage at the stage DN3 and severe thymus atrophy

(**A** and **B**) Bar graphs representing absolute numbers of thymocyte shown in Figure 1, C (n= 6, 4). (**C**) H&E stain of paraffin-embedded thymus sections from control (*Ada*$^{+/+}$) and untreated *Ada*$^{-/-}$ mice at P14. Scale bar is 1 mm. DN = double negative, DP= double positive, SP= single positive. C= cortex, M= medulla. *P <0.05, **P <0.01 and ***P <0.001. NS= statistically not significant.

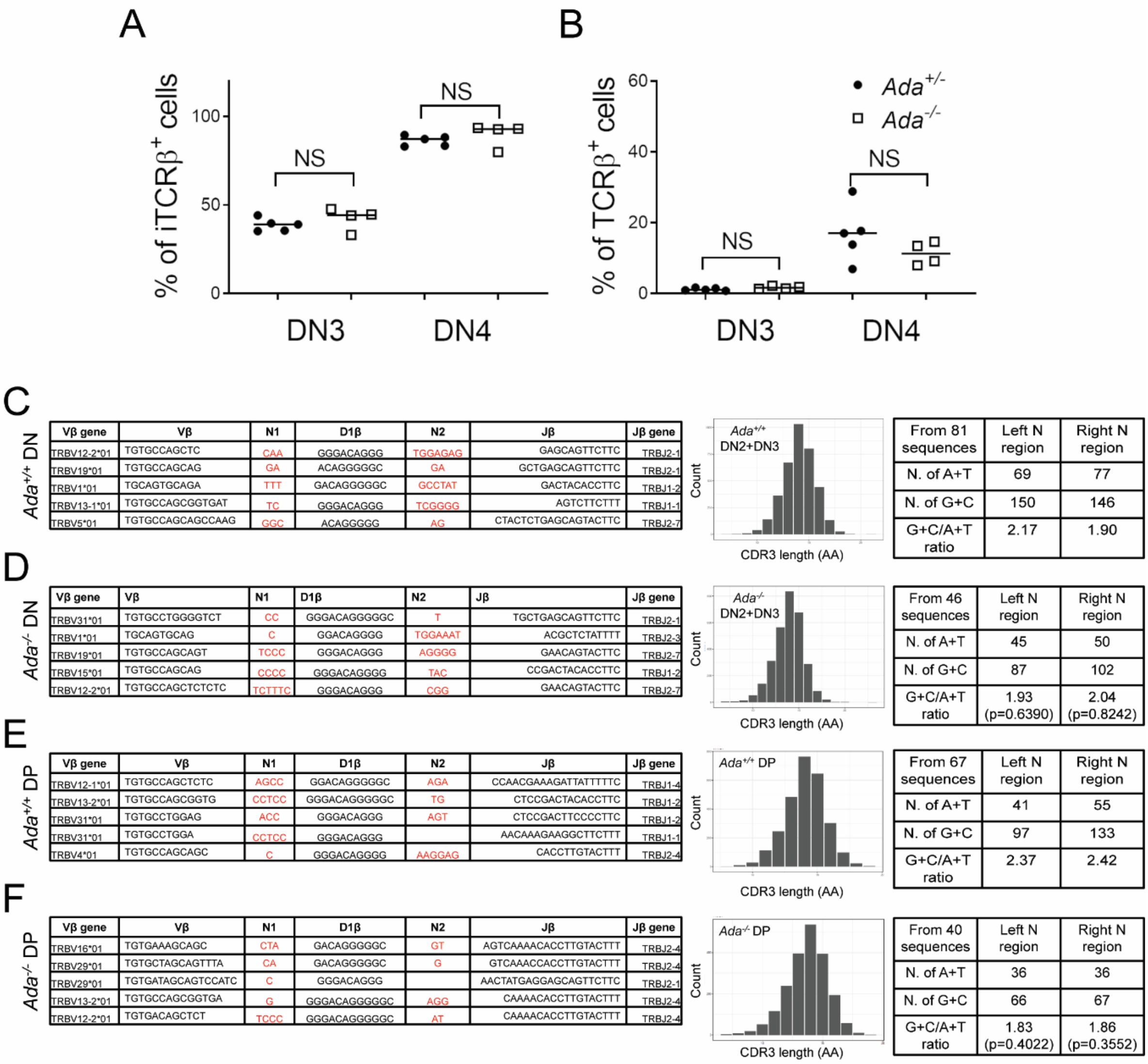

G

Cell types	*No. of nucleotides in N1 region (nt)*	*Mean insertion size in N1 region (bp)*	*No. of nucleotides in N2 region (nt)*	*Mean insertion size in N2 region (bp)*	*No. of clonotypes analyzed (sequences)*
Ada+/+ DN (CD25+)	0-12	2,7	0-11	2,8	81
Ada+/+ DP	0-12	2,1	0-13	2,8	67
Ada-/- DN (CD25+)	0-12	2,9	0-15	3,3	46
Ada-/- DP	0-12	2,6	0-8	2,6	40
All samples combined	0-12	2,5	0-15	2,8	234

Figure S5. ADA deficiency and nucleotide pool imbalance do not induce alterations of N-region insertions during V(D)J recombination

Percentage of DN3 (Lin^{neg}, CD44^{-}, CD25^{+}) and DN4 (Lin^{neg}, CD44^{-}, CD25^{+}) cells expressing TCRβ intracellularly (iTCRβ) (**A**) or on the cell surface (**B**) in control (*Ada*$^{+/-}$) and untreated *Ada*$^{-/-}$ mice at P14

(n= 5, 4). Statistical analysis was performed using the two-tailed homoscedastic Student's t-Test. NS= statistically not significant. Plots in (A) and (B) were generated by GraphPad Prism 7 software. (**C-F, left panels**) Example DNA sequences of the V, D, J and N regions in the TCRβ chain CDR3 of FACS-sorted DN2+DN3 (Lin^{neg}, $CD25^+$) (C and D) and DP ($CD4^+$, $CD8^+$) (E and F) cell populations from control (*Ada*$^{+/+}$) (C and E) and untreated *Ada*$^{-/-}$ (D and F) mice at P14. (**C-F, middle panels**) Length distribution of CDR3 regions, based on amino acid number, of *Ada*$^{+/+}$ DN2+DN3 (C), *Ada*$^{-/-}$ DN2+DN3 (D), *Ada*$^{+/+}$ DP (E), *Ada*$^{-/-}$ DP (F) cell populations. (**C-F, right panels**) Ratio of G+C/A+T contents in the N regions (red bases) of *Ada*$^{+/+}$ DN2+DN3 (C), *Ada*$^{-/-}$ DN2+DN3 (D), *Ada*$^{+/+}$ DP (E), *Ada*$^{-/-}$ DP (F) cell populations where the full D gene could be identified. (**G**) Nucleotide number analysis of the N regions. Statistical analysis was performed using two-tailed Fisher's exact test. P values <0.05 were considered statistically significant. CDR3= complementarity-determining region 3, AA= amino acid.

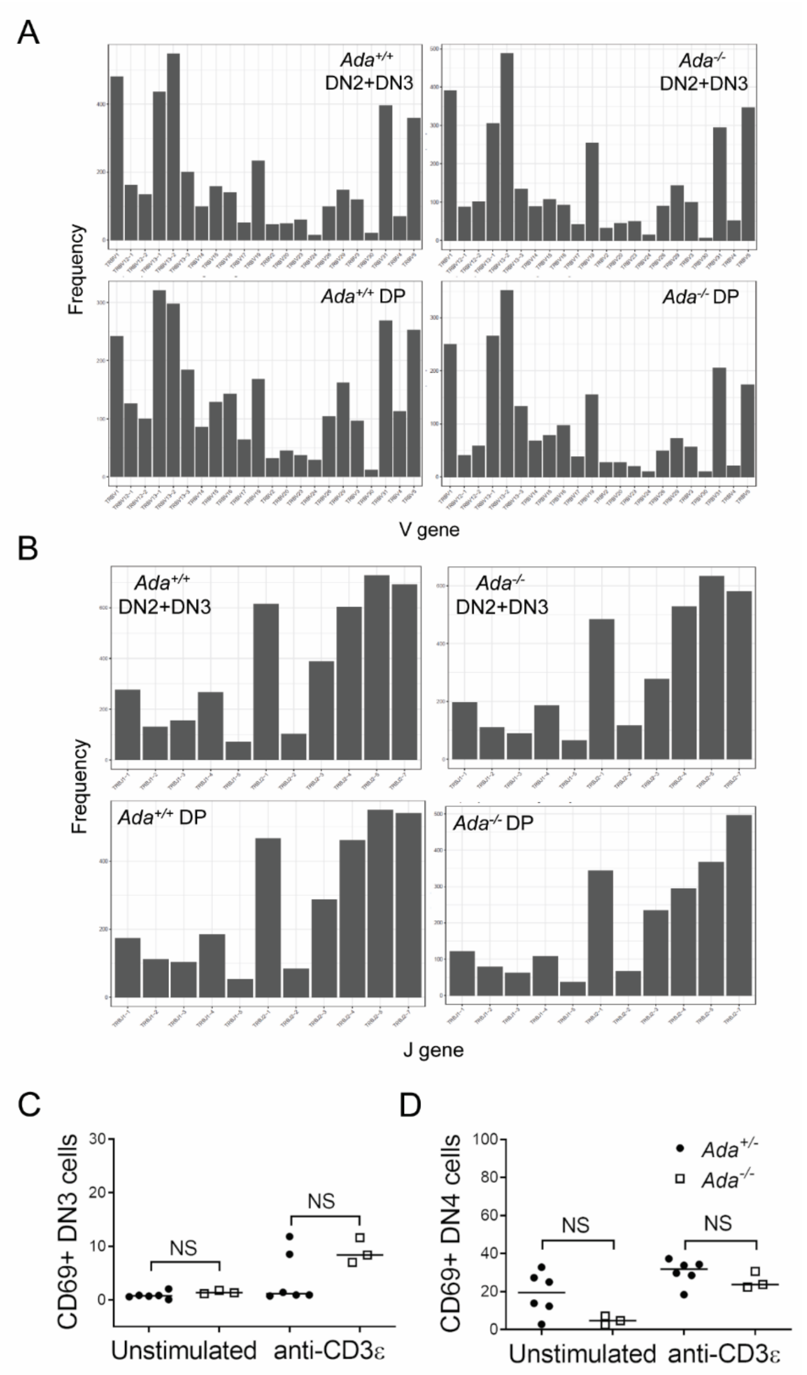

Figure S6. ADA deficiency does not alter the usage frequency of V and J elements during V(D)J recombination

Frequency distribution of V (**A**) and J (**B**) genes usage in *Ada*$^{+/+}$ DN2+DN3 and DP (A and B, left panels) and in *Ada*$^{-/-}$ DN2+DN3 and DP (A and B, right panels) cell populations. (**C**) Percentage of DN3 (Lin^{neg}, CD44^{-}, CD25^{+}) and (**D**) DN4 (Lin^{neg}, CD44^{-}, CD25^{+}) thymocytes expressing CD69 on the cell surface in control (*Ada*$^{+/-}$) and untreated *Ada*$^{-/-}$ mice at P14. Tested cells were either left untreated (n= 6, 5) or stimulated (n= 6, 3) with immobilized anti-CD3e Ab. Plots in (C) and (D) were generated by GraphPad Prism 7 software. NS= not significant.

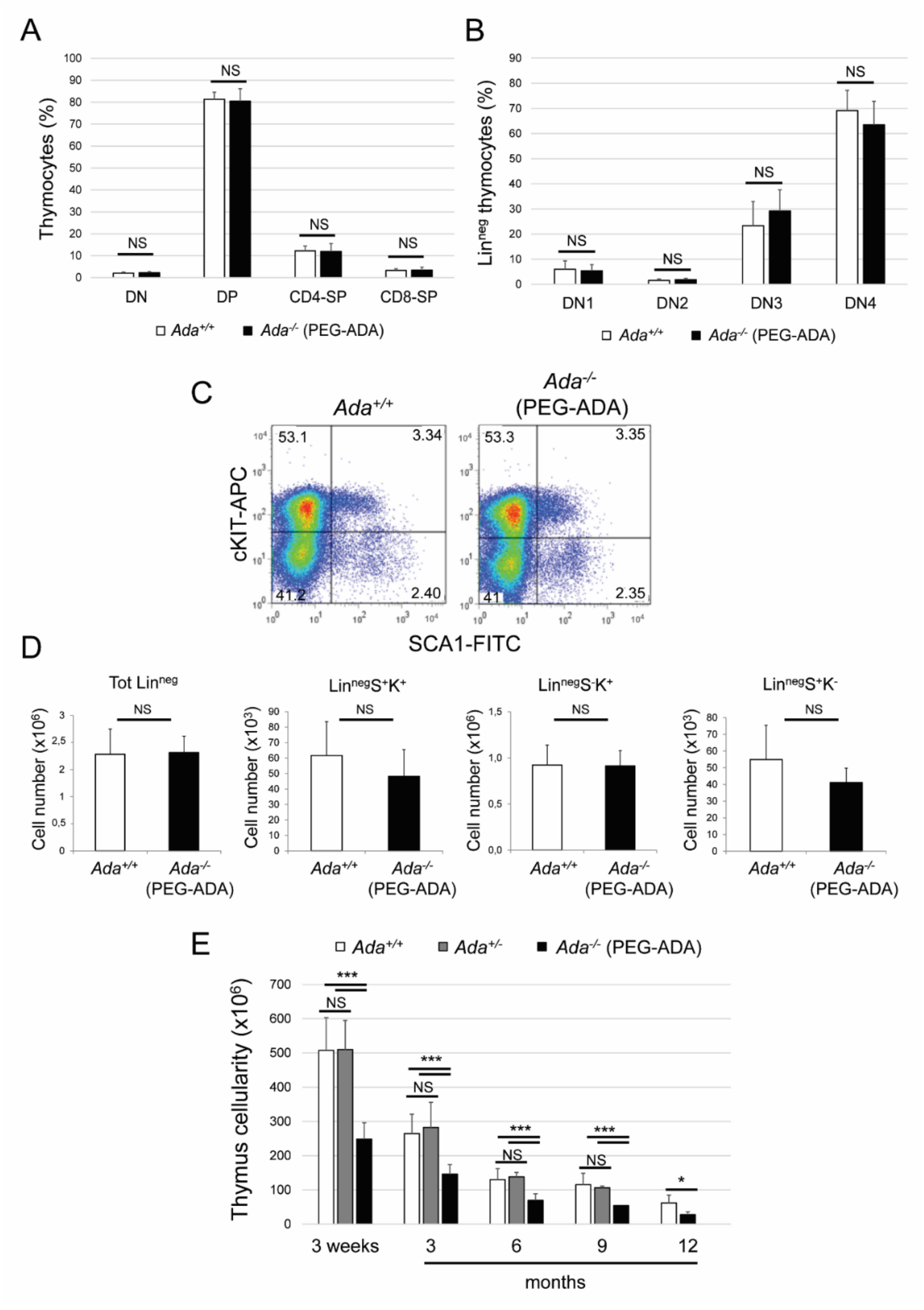

Figure S7. Enzyme replacement therapy normalizes hematopoietic stem cell numbers but does not normalize thymocyte numbers in ADA-deficient mice

(**A** and **B**) Bar graphs representing percentages of cell populations shown in Figure 4, D and E (n= 6, 7). (**C**) FACS plots of BM lineage negative (Lin^{neg}) cell populations from control (*Ada*$^{+/+}$) and 3-month-

PEG-ADA-treated *Ada*$^{-/-}$ mice: primitive hematopoietic stem cells (SCA1^{+}, cKIT^{+}), myeloid progenitor cells (SCA1^{-}, cKIT^{+}) and early lymphoid-committed precursors (SCA1^{+}, cKIT^{-}). (**D**) Bar graphs representing absolute numbers of cell populations shown in panel C per mouse (n= 7, 6). (**E**) Cellularity of thymi from control (*Ada*$^{+/+}$, *Ada*$^{+/-}$) and PEG-ADA-treated *Ada*$^{-/-}$ mice at different time points (3 weeks, n= 5, 3, 6; 3 months, n= 10, 9, 13; 6 months, n= 12, 3, 14; 9 months, n= 9, 4, 12; 12 months, n= 4, 5). *P <0.05, **P <0.01 and ***P <0.001. NS= statistically not significant.

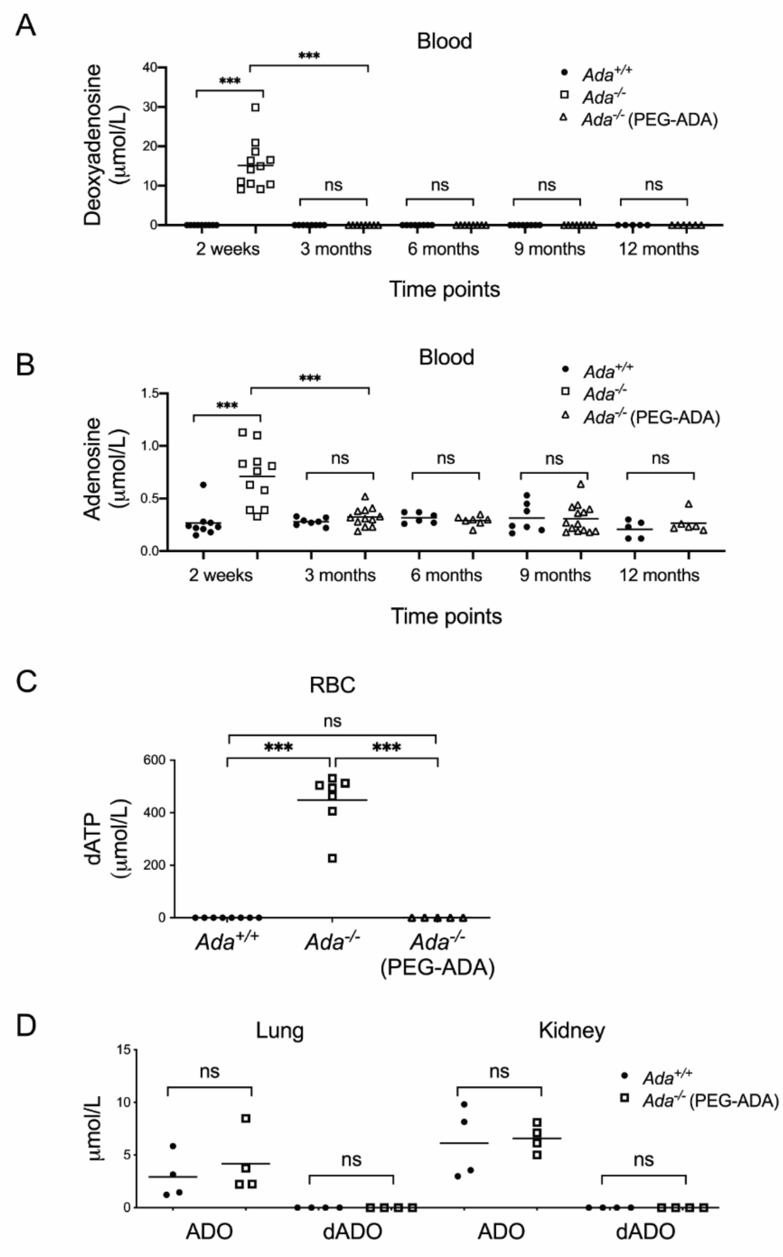

Figure S8. Enzyme replacement therapy normalizes purine metabolite levels in blood, lung and kidney

Tandem MS measurement of (**A**) dAdo (2 weeks, n= 9, 12; 3 months, n= 8, 8; 6 months, n= 8, 8; 9 months, n= 8, 8; 12 months, n= 5, 6) and (**B**) Ado (2 weeks, n= 9, 11; 3 months, n= 7, 12; 6 months, n= 6, 7; 9 months, n= 7, 14; 12 months, n= 5, 6) levels in blood from control (*Ada*$^{+/+}$), untreated and PEG-ADA-treated *Ada*$^{-/-}$ mice at different time points. (**C**) Reversed phase HPLC measurement of dATP levels of RBC from control (*Ada*$^{+/+}$), untreated (2 weeks) and PEG-ADA-treated *Ada*$^{-/-}$ (6 months) mice (n= 7, 7, 5). (**D**) Tandem MS measurement of Ado and dAdo levels in lung and kidney from control (*Ada*$^{+/+}$) and 3-month-PEG-ADA-treated *Ada*$^{-/-}$ mice (n= 4, 4). All plots were generated by GraphPad Prism 7 software. *P <0.05, **P <0.01 and ***P <0.001. NS= statistically not significant.

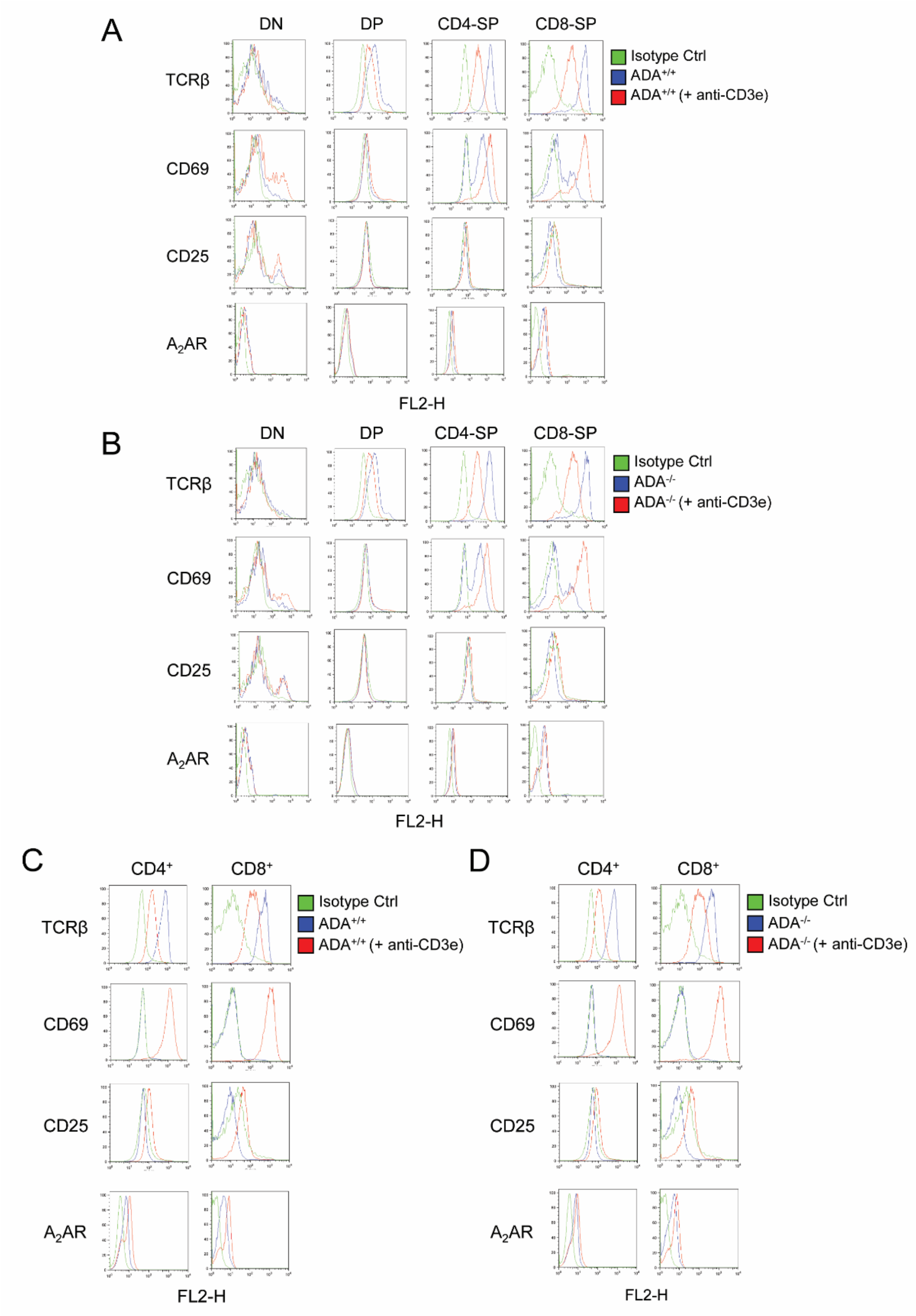

Figure S9. ADA-deficiency does not alter T cell activation in thymus and spleen

FACS analysis of surface receptor expression in thymocyte (**A** and **B**) and T splenocyte (**C** and **D**) populations from control (*Ada*$^{+/+}$) (**A** and **C**) and 3-week-PEG-ADA-treated *Ada*$^{-/-}$ (**B** and **D**) mice. Tested cells were either left untreated (n= 2; 2) or stimulated (n= 2; 2) with immobilized anti-CD3e Ab.

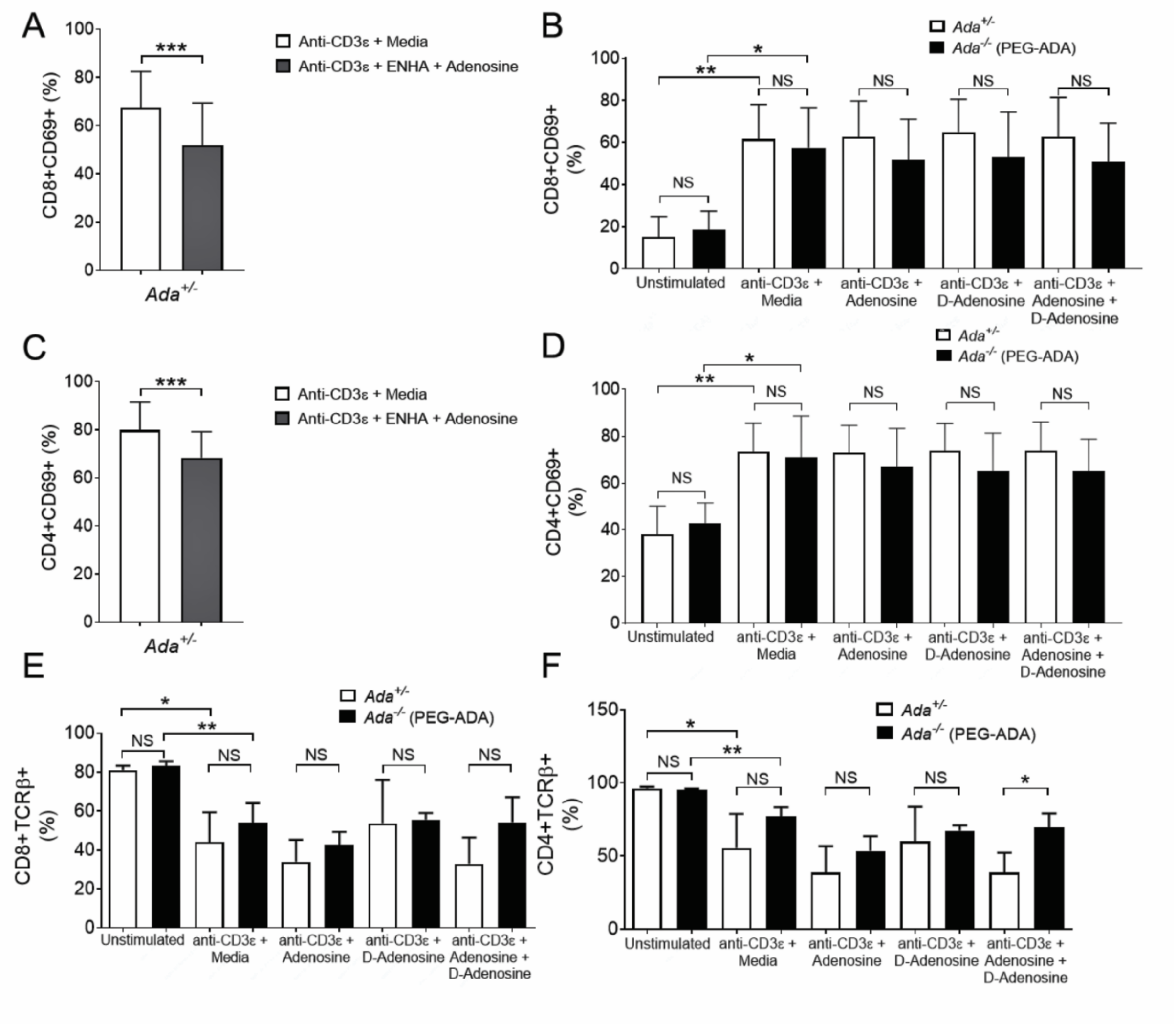

Figure S10. ADA-deficiency does not alter surface receptor expression in thymocytes

Bar graphs representing percentages of surface receptor expression on $CD8^+$ (**A, B** and **E**) and $CD4^+$ thymocytes (**C, D** and **F**) in different culture conditions. Tested cells were either left untreated or stimulated with immobilized anti-CD3e Ab. Thymocytes were obtained from control (*Ada*$^{+/-}$) and 3-week-PEG-ADA-treated *Ada*$^{-/-}$ mice. (A and C, n= 6, 6), (B and D, n= 4, 4), (E and F, n= 3, 3). We used two-tailed paired (A and C) and two-tailed homoscedastic (B, D, E and F) Student's t-Test *P <0.05, **P <0.01 and ***P <0.001. NS= statistically not significant.

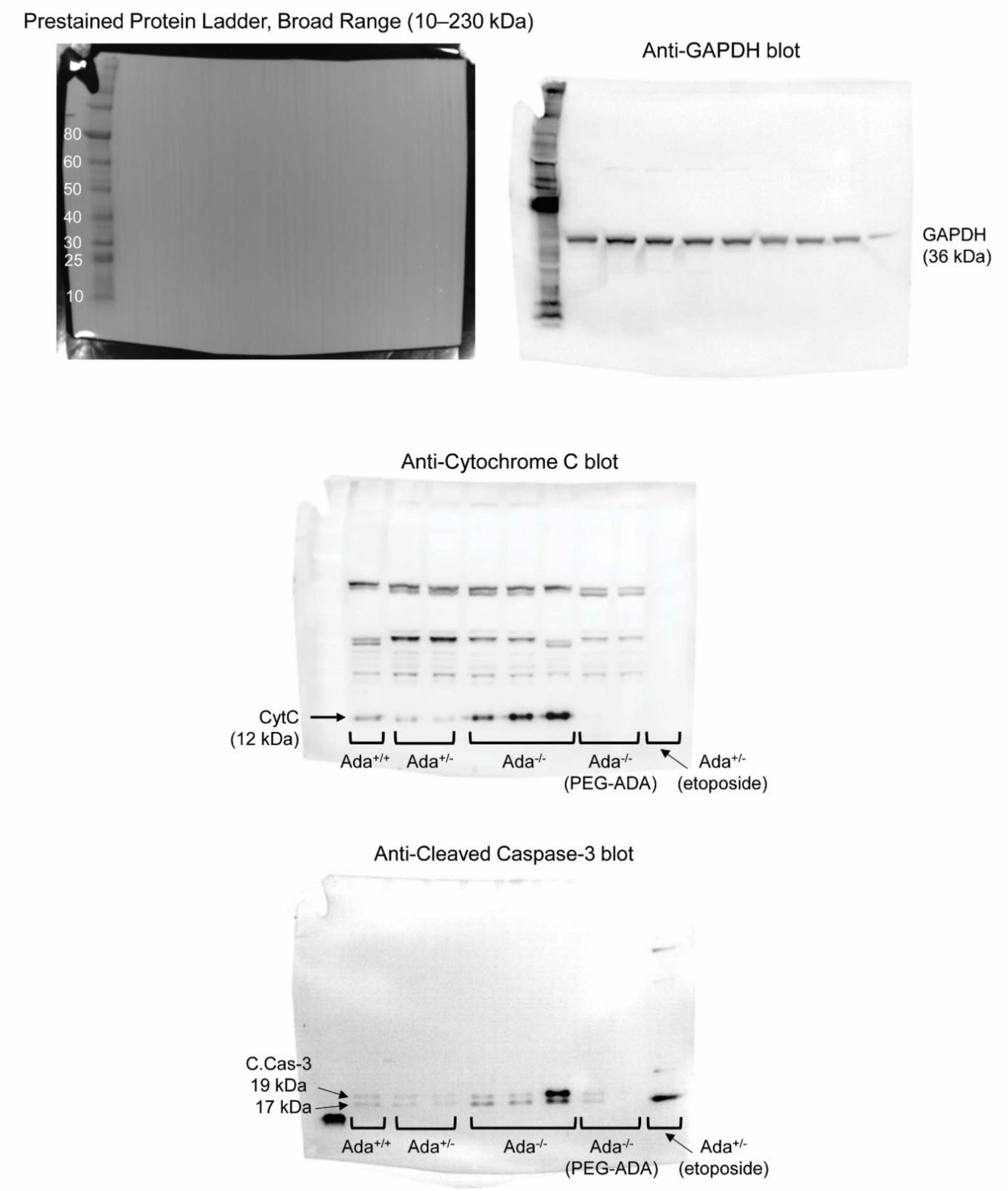

Figure S11. Full-length Western blots of the Figure 3A.

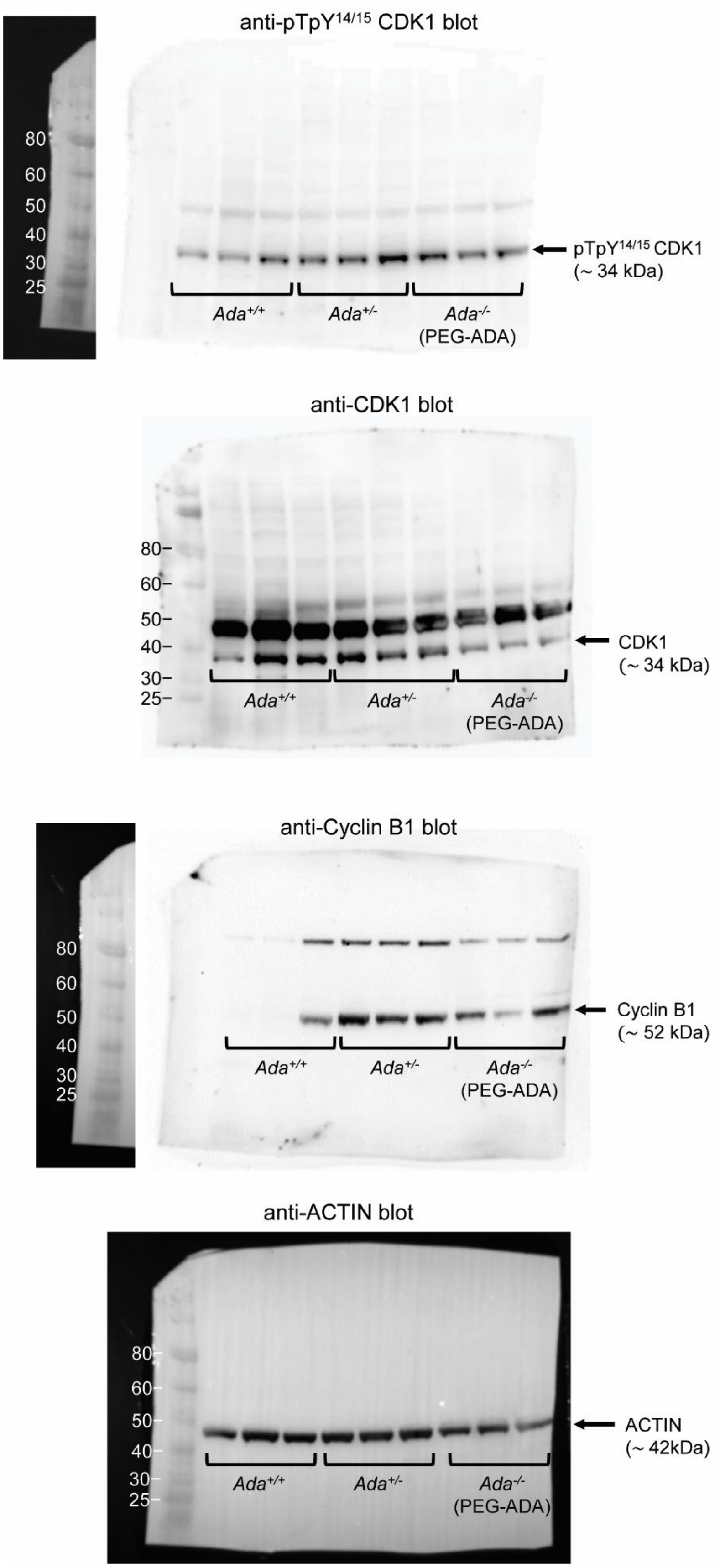

Figure S12. Full-length Western blots of the Figure 7E.

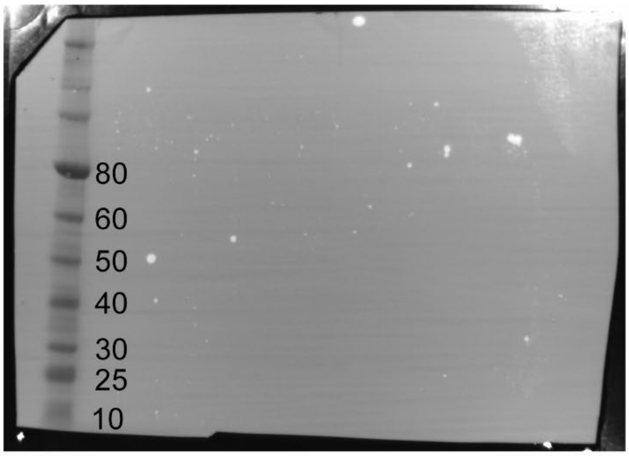

(Sheep) anti-mouse ADA blot

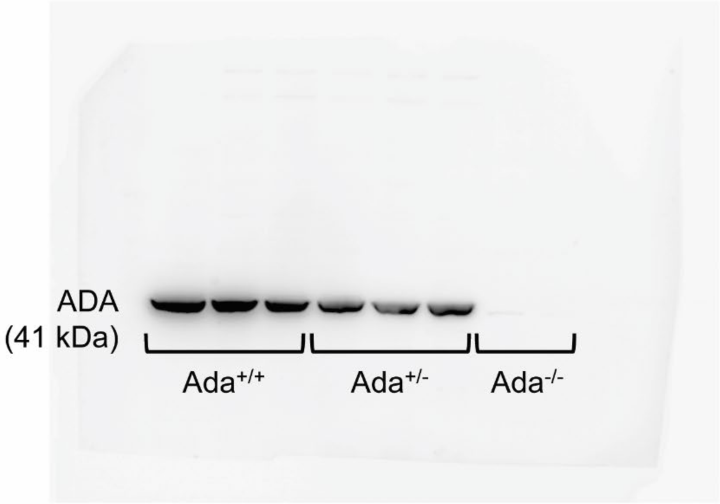

(Rabbit) anti-mouse GAPDH blot

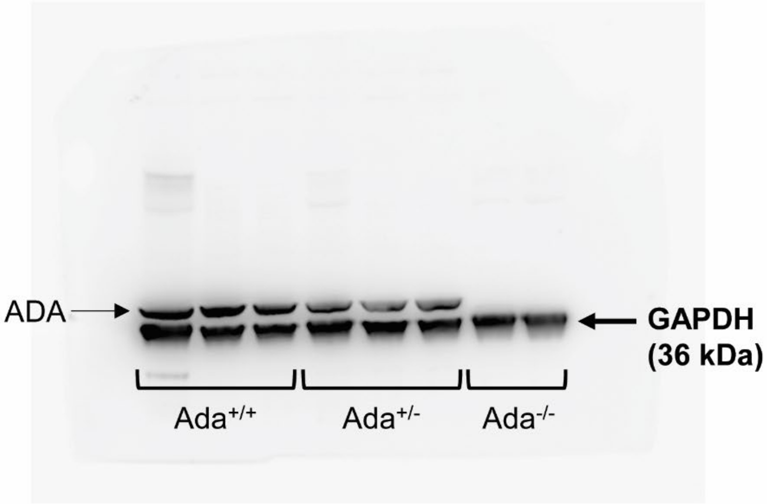

Figure S13. Full-length Western blots of the Figure S1A.

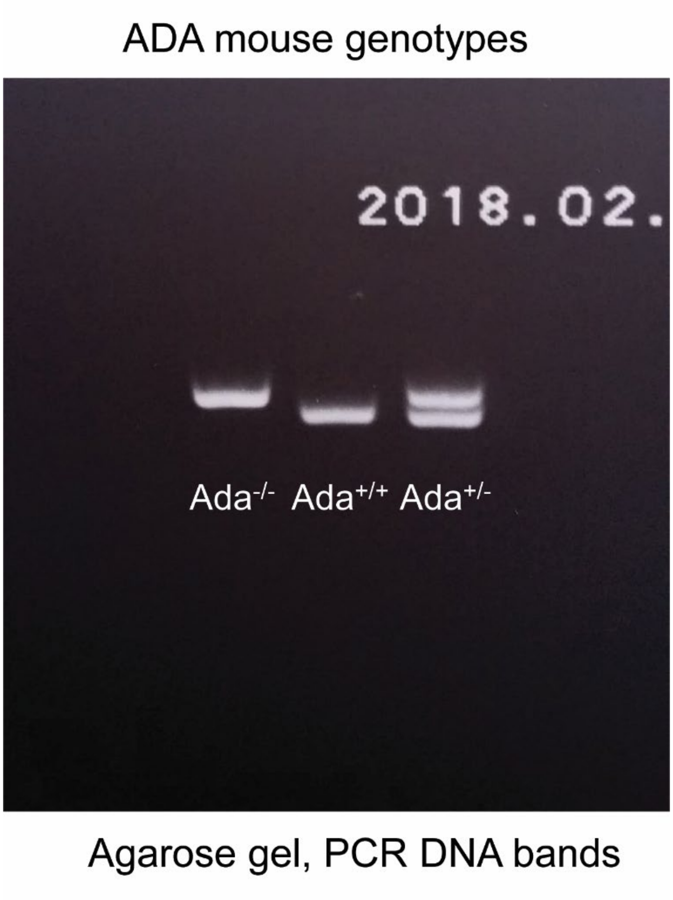

Figure S14. Full-length Agarose gel of the Figure S1G.
